# What could be? Depends on who you ask: Using latent profile analysis and natural language processing to identify the different types and content of utopian visions

**DOI:** 10.1111/bjso.12853

**Published:** 2025-02-03

**Authors:** Morgana Lizzio‐Wilson, Emma F. Thomas, Michael Wenzel, Emily Haines, Jesse Stevens, Daniel Fighera, Patrick Williams, Samuel Arthurson, Danny Osborne, Linda J. Skitka

**Affiliations:** ^1^ Department of Psychology University of Exeter Exeter UK; ^2^ College of Education, Psychology, and Social Work Flinders University Adelaide South Australia Australia; ^3^ School of Psychology University of Auckland Auckland New Zealand; ^4^ Department of Psychology University of Illinois at Chicago Chicago Illinois USA

**Keywords:** collective action, latent profile analysis, natural language processing, prospection, utopian thinking

## Abstract

When people think of a utopian future, what do they imagine? We examined (a) whether people's self‐generated utopias differ by how much they criticize, seek to change or escape from an undesirable present; and (b) whether these distinct types of utopian thinking predict system‐critical attitudes and intentions to change the status quo. Participants (*N* = 509) wrote about a future where a social issue they supported was resolved (e.g. economic inequality and climate change). Latent profile analysis revealed a subgroup of *change‐oriented utopian thinkers* with lower system satisfaction and higher action intentions than the other two subgroups. Unexpectedly, the remaining profiles imagined ominous (*dystopian thinkers*) or ‘neutral’ (*ambivalent future thinkers*) futures and expressed mixed social change support. Computerized linguistic analyses further revealed that dystopian thinkers used more hopelessness‐related language than change‐oriented utopian thinkers. Ambivalent future thinkers were as ‘hopeless’ as dystopian thinkers but, like change‐oriented utopian thinkers, used more fairness‐related language. Thus, change‐oriented utopian thinkers distinctly imagined a fairer—and possible—future. These results illustrate heterogeneity in how people imagine the future of their societies on specific issues. Critically, the features of these visions predict system‐critical attitudes and a willingness to agitate for change.

## INTRODUCTION

I have a dream that one day this nation will rise up and live out the true meaning of its creed: We hold these truths to be self‐evident, that all men are created equal. –Martin Luther King Jr

People often imagine ideal future possibilities when pursuing social change (Sweetman et al., 2013). Rather than fleeting products of the imagination or idle dreams, these visions can be an important precondition to critiquing and challenging the status quo (Solnit, 2016). Indeed, *utopian thinking* (i.e. the process of imagining ideal, future societies; Fernando et al., 2018) inspires people to act by helping them conceive of how things could be different and how to work towards specific changes (Kashima & Fernando, 2020).

Levitas (2013) proposed that utopias serve three functions. Utopias can not only encourage *criticism* of, and a desire to, *change* the system but they can also allow people to *compensate* or escape from an undesirable present and, in doing so, diminish efforts to challenge the status quo (Fernando et al., 2018). Given these diverging consequences, it is important to understand when imagining ideal futures will foster societal (dis)engagement (Kashima & Fernando, 2020). Indeed, utopian thinking does not always cultivate criticism and change. For example, Bird, Thomas, Wenzel, and Lizzio‐Wilson (2024) found that utopian thinking reduced exhaustion among climate justice supporters, but did not influence collective action. Thus, imagining an ideal future helped participants cope with negative emotions but failed to promote actions to address the source of the problem. Similarly, Daysh et al. (2024) found that utopian thinking did not consistently directly affect collective climate action. Instead, imagining an ideal future simultaneously enhanced hope that change might happen and lowered negative emotions (fear) that create the impetus to act for change. Consistent with Levitas's (2013) intuition, the current literature suggests that positive, future‐oriented thought can motivate both engagement (via criticism, change) and disengagement (via compensation; see Kashima & Fernando, 2020). But when or why it does so is not yet well understood.

One way in which these processes may be better understood is by examining the *content or features of people's self‐generated future visions*. Fernando et al. (2020) showed that the content of imagined futures influenced their motivational potential, such that ‘green’ utopias which embodied sufficiency (i.e. balancing human comfort with ecological sustainability) motivated stronger change motivation than ‘sci‐fi’ utopias that embodied maximization (i.e. increasing levels of leisure and material comfort).

Building on Fernando et al. (2018, 2020), we examined whether the features of people's self‐generated utopias affect their motivational potential. We explored: (a) if people's self‐generated utopias differ in the extent to which they depict the future as desirable, beneficial for the greater good, innovative and possible; and (b) if different types or combinations of these features may be relevant in understanding the criticism, change and compensation functions of utopias, reflected in differences in system critical attitudes (system justification, anger and moral convictions) and a willingness to change the status quo (hope, group efficacy, opinion‐based identification and collective action intentions).

### The motivating potential of different utopias

Although previously conceptualized as consequences of utopian thinking, the extent to which people's self‐generated futures highlight shortcomings (*critical utopian thinkers*), strive to change the status quo (*change‐oriented utopian thinkers*) and/or seek to escape from present circumstances (*escapist utopian thinkers*) could predict differences in their social change support. That is, the extent to which criticism, change and compensation are characteristic of people's self‐generated utopias in and of themselves may differentially predict system critical attitudes and willingness to agitate for change. To examine the heterogeneity of self‐generated utopias, we must adopt an approach that can identify relevant subgroups of people who imagine their futures in similar ways. Given that utopias can differ on multiple dimensions and that our theorizing suggests that these are properties of some groups of people relative to others, we adopted a *person‐centred approach* (Osborne & Sibley, 2017): Latent Profile Analysis (LPA).

Although prior work has found that the three functions of utopias are positively correlated (Fernando et al., 2018), these associations have been observed when taking a *variable‐centred approach*—an approach that assumes that participants are collected from a uniform population (Thomas et al., 2024). However, this does not preclude the possibility that there are subgroups of people whose utopias embody these functions to a greater or lesser extent and who, consequently, differ in their social change support. In adopting person‐centred methods, we can model the heterogeneous responses people may have when asked to construct their future visions. In contrast, variable‐centred methods cannot always capture these complex responses, which may obscure or nullify any direct relationships or associations with outcomes of interest (e.g. Bird, Thomas, Wenzel, & Lizzio‐Wilson, 2024; Daysh et al., 2024; see also Lizzio‐Wilson et al., 2021, for an example in the context of collective action). Thus, adopting a person‐centred approach can identify if there is indeed heterogeneity in people's self‐generated utopias, and if there are certain ‘types’ of utopian visions which predict greater social change support.

Notably, LPA is inherently exploratory and inductive. The analysis is repeated with increasing numbers of profiles and the optimal solution is determined based on statistical and theoretical fit. Nevertheless, our approach was guided by theoretically derived expectations about the likely number of profiles that will emerge (see Lizzio‐Wilson et al., 2021, 2023; Osborne et al., 2024; Yip et al., 2025 for a cognate approach). The final solution may, however, be different to the profiles theorized below.

### Criticism, change and compensation as different types of utopian thinking

We explored how people's self‐generated utopias differ along four dimensions (*desirable*, *beneficial for the greater good*, *innovative* and *possible*) which may collectively reflect the extent to which criticism, change or compensation/escapism are characteristic of their imagined future. Like utopian thinking, social movements are predicated on actualizing an ideal, imagined future (Hawlina et al., 2020). However, other psychological factors must coalesce in conjunction with these ideals for people to critique and challenge the status quo. Specifically, people not only need to know *what* social good they are working towards (Hawlina et al., 2020), but also concretely envision *how* the status quo should be re‐ordered (Turner & Brown, 1978) and believe that change *might be* achievable (Bury et al., 2016, 2020).

We propose that these dimensions may also be important features of utopian visions and differentiate people who construct utopias that encapsulate criticism of the status quo, a desire to challenge the current system and escape from discomfort. As we argue below, social change motivation may not be evidenced by a desirable future alone, but a future that also describes a *social good* to work towards (beneficence), *how* change can occur (innovation) and *if* those changes could happen (possibility).

#### Desirable

Although utopian thinking inherently involves imagining ideal future societies (Kashima & Fernando, 2020), desirability may be a necessary, but not sufficient, precondition for social change support. People engage in desirable activities to cope with and escape stress (Stenseng et al., 2012). Doing so may lessen any negative emotional effects and provide a distraction but does not promote actions to address the source of the stressor (Meier et al., 2018; Wulf et al., 2022). Thus, when thinking about the future, people may need something positive to work towards, but other features are needed *in conjunction with* desirability to produce critique and change. Otherwise, envisioning an ideal future may help some escape from undesirable circumstances without accompanying social change support (Bird, Thomas, Wenzel, & Lizzio‐Wilson, 2024).

#### Beneficence

An important differentiating feature, however, may be the extent to which people indicate that their utopias benefit the greater good. This dimension encapsulates pro‐social actions and attitudes that seek to benefit others and promote their welfare and rights (Batson, 1991). Although beneficence is another positive aspect of utopian thought, it may be a unique feature of critical and/or change‐oriented utopias because it reflects a critical examination of how future societies can function as a social good. When imagining future worlds, people are more likely to support social change (e.g. policy changes, collective action) if they believe that their imagined future would reduce societal dysfunction (e.g. inequality, Judge & Wilson, 2015) and increase societal development and morality (Bain et al., 2013; Milfont et al., 2014). Thus, imagining ‘how good and just things could be’ implies a critical examination that the future can and should improve upon the present, and predicts attitudes and intentions to actualize these possible realities. Based on this reasoning, we expect that beneficence will be higher among critical and change‐oriented thinkers than escapist utopian thinkers. While escapist utopian thinkers may construct futures that are desirable, they do not encapsulate improvements to the status quo in terms of promoting others' rights and welfare.

#### Innovative

Believing that change *should* happen does not mean that people are able to imagine *how* change will happen. Thus, a unique feature of change‐oriented utopian thinkers may be their ability to imagine specific ways the status quo can be changed (Reicher & Haslam, 2006). This could be evidenced by higher innovation, which captures the extent to which people can freely imagine new and potentially radical alternatives to the status quo, and specific ways that contemporary social problems might be resolved (Badaan et al., 2020; Sargisson, 2000). Because social change often requires major societal transformations, being able to concretely envisage how current social systems might be reordered may provide a blueprint for transformational change and a concrete set of goals to work towards (Bosone et al., 2024; Wright et al., 2020, 2022). Thus, change‐oriented utopian thinkers might be characterized by higher innovation because they can imagine a desirable future that increases societal development (desirability, beneficence) and *how* these changes might be achieved.

In contrast, innovation may be lower among critical utopian thinkers. Although they are dissatisfied with the present, they are less able to concretely imagine alternative realities that improve upon the present (Turner & Brown, 1978). We had competing expectations about the role of innovation for escapist utopian thinkers. It could be that this profile will report higher innovation. Because their utopias do not seek to benefit the greater good, innovation (coupled with desirability) may reflect an escape into a ‘fantasy world’ (Mandel & Smeesters, 2008), rather than formulating creative solutions to transform the status quo. Alternatively, it could also be that escapist utopian thinkers will report lower innovation because, like critical utopian thinkers, they cannot imagine *how* the status quo could be changed.

#### Possible

Social change is rarely certain or directly controllable. As such, success is not always predictable or probable (Bruininks & Malle, 2005). However, the belief that a desired outcome is possible (rather than probable) can inoculate people against resignation and motivate actions to facilitate change even when ‘the odds are against them’ (Bury et al., 2016, 2020). As such, change‐oriented utopian thinkers may indicate that their utopias are more possible than the other profiles because their imagined futures reflect a truly ideal society (in terms of desirability, beneficence and innovation), coupled with an investment in the possibility that this vision could be actualized. But without ‘investing in the possibility’ that change might happen, people can psychologically escape their discomfort (Zeigler‐Hill et al., 2024) or remain critical of the status quo while perceiving inequality as enduring (Bird, Thomas, & Wenzel, 2024).

### Predicting differences in system critical attitudes and willingness to agitate for change

Each type of utopian thinking should differentially predict outcomes^1^ that reflect criticism of the status quo (i.e. system justification, anger, moral convictions about how the world should be), and a belief in the ability to change the current system and willingness to take action to do so (i.e. hope, group efficacy, opinion‐based identification and collective action intentions).

#### Criticism‐related outcomes: dissatisfaction with the status quo

We expect that critical utopian thinkers will report lower satisfaction with the status quo relative to the other profiles (i.e. lower system justification, higher anger and stronger moral convictions). Consistent with this idea, Fernando et al. (2018) found that utopian thinking decreased endorsement of system justification (i.e. perceiving the current social system as fair; Jost et al., 2004), suggesting that thinking about an ideal future can highlight the failings of current social structures. Similarly, people experience anger in response to perceived unfairness (Weiss et al., 1999) which predicts dissatisfaction with the status quo (Whitson et al., 2015). Thus, critical utopian thinkers may be least inclined to justify current social systems, and most likely to experience emotions that reflect negative appraisals of the existing situation.

Critical utopian thinkers may also report stronger *moral convictions*, which reflect a meta perception that one's attitude reflects beliefs about right and wrong (Skitka, 2010). Unsurprisingly, people react negatively when their moral convictions are violated because there is a disparity between ‘how things are’ and ‘how they should be’ (Mullen & Skitka, 2006). Because critical utopian thinkers' imagined futures highlight how the present (vs. an ideal future) violates important moral values (e.g. about justice and equality), making this disparity salient may prompt them to moralize their attitudes about a particular social issue relative to the other profiles (Wisneski & Skitka, 2017).

#### Change‐related outcomes: willingness to challenge the status quo

Although critical utopian thinkers may question traditional status hierarchies, their utopias could lack two ‘ingredients’ that may undermine their willingness to challenge the status quo: (a) innovative alternatives that provide a blueprint for social change (innovation), and (b) the belief that their imagined future is possible. Change‐oriented utopian thinkers, however, should be higher in both ingredients than those in the other profiles. Thus, change‐oriented utopian thinkers *should* report higher change‐related outcomes than the other profiles (i.e. higher hope, group efficacy, opinion‐based identification and collective action intentions).

*Hope* is a future‐oriented emotion that arises when thinking about a desired future outcome and involves generating alternatives to compare against present circumstances (Bury et al., 2020; Thomas et al., 2022). Thus, hope is a positive emotion that pairs positive feelings about the future with the belief that things can change (Lazarus, 1991, 1999). Similarly, *group efficacy* is the belief that change is possible in united effort with others and is an important predictor of action towards the achievement of collective goals (van Zomeren et al., 2008). Because change‐oriented utopian thinkers construct utopias that act as a goal towards which they strive to change the status quo, they will likely report stronger emotional (hope) and cognitive (group efficacy) reactions than other profiles of utopian thinkers, reflecting their belief.

*Identifying as a supporter of change* is also an important indicator of change commitment. Identification with groups who share people's views about how the world should be (i.e. opinion‐based groups; Bliuc et al., 2007; McGarty et al., 2009) enable group members to collectively express their stance on a particular issue and challenge the status quo. Because people can come to identify with groups via a sense of dissatisfaction about the status quo and/or the belief that collective efforts successfully address injustice (Thomas et al., 2012, 2016), critical and change‐oriented utopian thinkers may report higher opinion‐based identification than escapist utopian thinkers.

Finally, change‐oriented utopian thinkers may be more willing to *engage in collective action*. Because this profile will perceive their imagined future as more likely, this should help inoculate against resignation and spur their willingness to engage in action to achieve this idealized society (Bury et al., 2020).

### The present research

We sought to examine potential differences in people's social change commitment across different types of utopian visions. To test this, we asked supporters of three social issues (economic equality, climate change and gun control/rights) to imagine a future society where that specific issue had been resolved. Participants then rated the extent to which their self‐generated utopias were desirable, beneficial for the greater good, innovative and possible. In Phase 1, we used these responses to determine whether people's self‐generated utopias differed in the extent to which they criticize, seek to change or escape from the status quo; and whether these distinct types of utopias differentially predicted criticism‐ and change‐related outcomes (https://osf.io/cv4re/?view_only=28f94c57c29a4cc2868a79d88656db46). In Phase 2, we adopted a computerized natural language processing approach to analyse participants' written descriptions of their self‐generated futures. This was done to understand the content and psychological differences between profiles and explain their varied social change support (https://osf.io/6d327/?view_only=2c4ab544497d453aaf90a73b76217eac). Survey materials, data files and analysis code can be found here: https://osf.io/8sujz/?view_only=0b60de9553d64fd4912bd7f99d279880.

### Transparency statement

This project utilizes secondary data that were collected with the original purpose of experimentally examining the effects of utopian thinking on the criticism‐ and change‐related outcomes. Because there were few significant effects of the manipulation (see Data S1), we adopted a person‐centred approach to determine whether there is heterogeneity in people's self‐generated utopias, and if these differences influence the motivating potential of these future visions.

## PHASE 1

### Method

#### Participants and design

Participants were North American citizens recruited via Amazon Mechanical Turk in relation to one of three social issues: economic inequality (Study 1), climate change (Studies 2–3) and gun ownership regulation (Study 4). Participants in Studies 1–3 were all supporters of the issue in the given study. In Study 4, participants who supported gun control (i.e. stricter gun laws) and supported gun rights (i.e. more lenient gun laws) were both recruited and asked to reflect on an ideal future in relation to their stance on gun ownership regulation.

All studies employed between‐subjects experimental designs in which participants were randomly assigned to think about the future (vs. the present) in relation to a particular issue. Although some studies included other manipulations, these had few effects on the measures of interest (see Data S1). Because we sought to identify different types of utopian thinking, we only included participants who completed the utopian thinking exercise in each study.

Our final sample size was 509 (*M*_age_ = 39.94, SD = 12.57, 53.40% men, 46.40% women and 0.20% non‐binary). Table 1 summarizes the demographic information, design and number of participants retained from each study. Sample size calculations are not straightforward for LPA. Power is determined by the number of indicators, participants, and the composition and degree of separation between profiles which are all difficult to predict a priori (Tein et al., 2013). However, simulation studies suggest that a minimum *N* = 500 should provide sufficient power (on average) to accurately detect the number of latent profiles in a sample (Nylund et al., 2007; Spurk et al., 2020). Thus, our final sample size should provide sufficient power.

**TABLE 1:** Design and demographic information for each study.

	Study 1	Study 2	Study 3	Study 4
Social issue	Economic inequality	Climate change	Climate change	Gun ownership regulation
Total *N*	196	258	229	395
Design	2‐cell (time referent: present/future)	2 (future valence: positive/negative) × 2 (mental contrasting: present/absent) with a hanging control condition	2 (time referent: present/future) × 2 (group interaction: present/absent)	2 (time referent: present/future) × 2 (position: gun control/gun rights)
*n* in utopian thinking condition(s)	97	103	113	196
Demographic information for participants in the utopian thinking conditions				
Mean age (SD)	40.10^a^ (12.50)	38.18 (12.94)	37.42 (10.32)	42.22 (13.25)
Gender	50.50% female, 49.50% male	40.80% female, 59.20% male	51.30% female, 47.80% male, 0.90% non‐binary	44.40% female, 55.60% male

^a^ One participant did not provide their age.

#### Procedure and measures

Responses were assessed on a 1 (*strongly disagree*) to 7 (*strongly agre*e) Likert scale. Table 2 summarizes scale information for the measures used in each study.

**TABLE 2:** Scale information for the outcome measures included in each study.

Outcome	Study	α/*r*	Number of items	Example item
System justification	Study 1	.90	17	‘Most people who don't get ahead in our society should not blame the system; they have only themselves to blame’
	Study 2	.81	7	‘I find societal action on climate change to be fair’
	Study 3	.76	8	
	Study 4	.89	4	‘In general, the system operates as it should with regard to gun laws’
Moral convictions	Study 1	–	–	–
	Study 2	.92	3	‘My stance on climate change reflects my core moral values and convictions’
	Study 3	.86		
	Study 4	–	–	–
Anger	Study 1	.86	2	Anger, Outrage
	Study 2	.84		
	Study 3	.85		
	Study 4	.77		
Hope	Study 1	.92	2	Hope, Optimism
	Study 2	.89		
	Study 3	.86		
	Study 4	.79		
Group efficacy	Study 1	.88	2	‘Together supporters of efforts to reduce economic inequality can improve the outcomes for economically disadvantaged people in the United States’
	Study 2	.84		‘Together supporters of efforts to address climate change can improve the outcomes for everyone’
	Study 3	.73		
	Study 4	.82		‘Together, supporters of gun control [gun rights] can have a positive impact on how guns are regulated in the US’
Opinion‐based identification	Study 1	.96	3	‘I see myself as a supporter of efforts to reduce economic inequality [address climate change] (gun control/gun rights)’
	Study 2	.94		
	Study 3	.91		
	Study 4	.86		
Collective action intentions	Study 1	.93	7	‘I intend to contact politicians to express my support for action aimed at reducing economic inequality’
	Study 2	.89	7	‘I intend to reduce my own consumption of energy (i.e., ride bike more rather than drive)’
	Study 3	.92	9	‘I intend to divest my investments to those that promote climate change action’
	Study 4	.86	5	‘I intend to write or share a post on social media (e.g., Facebook or Twitter)’

*Note*: Reliability information is reported for the utopian thinking condition(s) in each study which were included in the final analyses. Cronbach's αs and *r*'s for the whole sample in each study can be found in the Data S1.

##### Utopian thinking task

After reading the information sheet and providing their informed consent, participants randomly assigned to the utopian thinking condition were asked to engage in a writing task (adapted from Fernando et al., 2018) in which they imagined and described an ideal future in which a specific social issue had been resolved (e.g. the climate crisis has been mitigated). Though the wording of the instructions varied slightly between studies (see OSF), all participants were asked to imagine and describe what this future would look like in a text box. This task was included to make a utopian society and utopian thinking salient to participants, and to provide context for the subsequent questionnaire. Participants were given 3–7 min to complete the task (depending on the study) before they could move forward.

##### Indicators of profile membership

Immediately after completing the utopian thinking task, participants were asked to rate the extent to which their imagined future was desirable (i.e. utopian, desirable, ideal; αs = .60–.92), beneficial for the greater good, innovative (i.e. imaginative, innovative, creative; αs = .87–.93) and possible. Although a different item was used to assess beneficence in Study 3 (i.e. prosocial), this alternative wording still encapsulates actions and attitudes that seek to benefit others and promote their welfare and rights (Pfattheicher et al., 2022).

##### Criticism‐ and change‐related outcomes

Participants then completed the outcome measures. Unless otherwise specified, all outcomes were assessed in each study using the same items. Table 2 contains example items.

###### System justification

Participants rated their agreement with a series of statements (4–17 statements per study) assessing the extent to which they perceived the current system in relation to their social issue as just and legitimate. Study 1 used the economic system justification scale (Jost & Thompson, 2000). Studies 2–4 used an adapted version of Kay and Jost's (2003) system justification scale.

###### Moral convictions

Three items (from Skitka et al., 2017) tapped the extent to which participants viewed the focal issue as a moral issue. These items were only included in Studies 2 and 3.

###### Anger and hope

Participants were asked to indicate the extent to which they felt anger (anger, outrage) and hope (hope, optimism) when thinking about the focal social issue.

###### Group efficacy

Two items (from Thomas et al., 2016) assessed the extent to which supporters of their specific cause felt they could effect change.

###### Opinion‐based identification

Three items (from Leach et al., 2008) measured the extent to which participants identified as supporters of efforts to address economic inequality, climate change or gun rights/gun control.

###### Collective action intentions

Participants indicated their intentions to engage in a series of actions (5–9 items per study) to address their specific social issue. While many of the actions were the same across each issue (e.g. signing a petition), some tactics were selected based on context‐specific (conventional) actions that were accepted and approved of within the real movement (see Louis et al., 2020).

### Results

#### Are there different types of utopian thinkers?

We first conducted an LPA on the profile indicators (desirable, benefit for the greater good, innovative and possible) using MPlus version 8.0. We used several metrics to assess model fit. Unlike cluster analysis, LPA incorporates a goodness‐of‐fit test for the number of subgroups in the data (McCutcheon, 2002; Osborne & Sibley, 2017). Thus, first, following Asparouhov and Muthén (2012), we compared a *k* − 1 model (where *k* = number of latent profiles) with a *k* model using the Vuong–Lo–Mendell–Rubin test (VLMR) and bootstrapped likelihood ratio test (BLRT). Second, we examined the Akaike (AIC), Bayesian information criterion (BIC) and adjusted BIC (aBIC) for each model, with smaller values reflecting a better fitting solution. Third, we considered model interpretability and fit with theory and model parsimony (e.g. by inspecting the profile plots; Muthén, 2006). Finally, we examined the entropy of each model, which captures the strength of separation between profiles. Values of >.80 indicate clear delineation between classes. Lower values do not indicate mis‐fit per se, but that the classes are less clearly distinguishable (Celeux & Soromenho, 1996). Table 3 summarizes the fit statistics for profile solutions with one to six profiles, entropy and the proportion of participants assigned to the different profiles.

**TABLE 3:** Model fit statistics for latent profile analysis comparing 1–6 profile solutions.

*k*	AIC	aBIC	BIC	VLMR, *p* value	BLRT, *p* value	Entropy	Size of profiles
Class 1	7147.485	7155.951	7181.344	–	–	–	100%
Class 2	6538.299	6552.057	6593.321	<.001	<.001	0.95	13%/87%
Class 3	6340.115	6359.165	6416.299	.008	<.001	0.90	6%/22%/72%
Class 4	6278.479	6302.820	6375.825	.111	<.001	0.84	5%/26%/7%/62%
Class 5^a^	5829.442	5859.075	5947.950	.051	<.001	1.00	5%/33%/13%/10%/39%
Class 6^a^	5422.182	5457.107	5561.853	.302	<.001	1.00	2%/5%/13%/39%/33%/8%

Abbreviations: AIC, Akaike; (a)BIC, (adjusted) Bayesian Information Criteria; BLRT, bootstrapped likelihood ratio test; VLMR, Vuong‐Lo–Mendell–Rubin likelihood ratio test.

^a^ Denotes that there was a problem with model non‐identification, and estimates may not be reliable.

Overall, these data supported a three‐profile solution. Reductions in the AIC, BIC and aBIC, and the significant VLMR and BLRT suggest that, empirically, a three‐profile solution provides a better fit to these data than does a two‐profile solution. Although the (conservative) BLRT suggested even more complex solutions were a better fit, the non‐significant VLMR and the relatively incremental changes in the AIC, BIC and aBIC for a four‐profile solution indicates that a three‐profile solution was most theoretically parsimonious without over‐extracting the data. The five‐ and six‐profile solutions were not identified, indicating that the solution was inadmissible and should not be interpreted (see Table 3).

**FIGURE 1:**
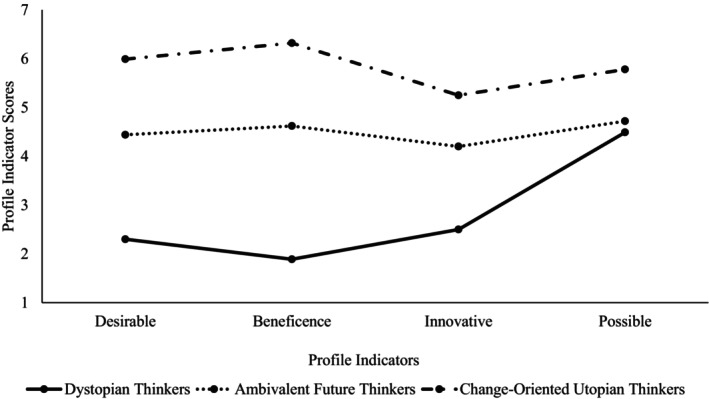
Line graph depicting the pattern of responses along the four profile indicators for the three latent profiles.

Figure 1 displays the mean values of the three profiles on each indicator. Most people (72%) belonged to a group we termed *changed‐oriented utopian thinkers*. This group closely aligned with one of the hypothesized profiles, as they reported that their self‐generated future was high on desirability and beneficence, and moderate‐to‐high on innovation and possibility. That is, they ‘agreed’ that their imagined future was desirable, beneficent, and might happen, and ‘somewhat agreed’ that it was a significant departure from the present.

Unexpectedly, the other profiles did not reflect the criticism or compensation functions of utopian thinking per se. A small proportion of the sample (6%) described future worlds that were low on desirability, beneficence and innovation, but were somewhat possible. That is, in absolute terms, they ‘disagreed’ that their imagined future was ideal, beneficent or a significant departure from the present, but were unsure if it could happen (i.e. they sat between ‘neither agree nor disagree’ and ‘somewhat agree’). Given that this profile described future worlds that were the antithesis of a utopia, we termed them *dystopian thinkers*.

Finally, the third profile (22%) sat between the other subgroups on each indicator: they ‘neither agreed nor disagreed’ that their future was desirable, beneficent or innovative, and ‘somewhat agreed’ that it was possible. We named this subgroup *ambivalent future thinkers* because their imagined futures were slightly above the mid‐point of the scale, suggesting that they were imagining a ‘neutral’ or less ambitious future that could be achieved. Thus, the LPA revealed the diverse ways that people construct the future when asked to envisage a utopia, rather than different types of utopian thinkers.

#### How do different types of utopian thinking predict criticism‐ and change‐related outcomes?

To assess differences in key outcomes across profiles, we used the distal three‐step approach that allows the predictive model to be estimated without affecting latent class formation (Asparouhov & Muthén, 2012). Table 4 summarizes the means and standard errors for each outcome variable across profiles. Though the final profiles differed from our expectations, we still conducted the mixture models to explore how the diverse ways that people think about the future predicted the criticism and change‐related outcomes.

**TABLE 4:** Means (standard errors) for outcome variables by profile membership.

	Dystopian thinkers	Ambivalent future thinkers	Change‐oriented utopian thinkers
System justification	3.75 (0.23)	3.91^a^ (0.12)	3.34^a^ (0.07)
Anger	5.05^b,c^ (0.27)	3.50^b^ (0.17)	3.50^c^ (0.10)
Moral convictions	5.22^b,c^ (0.23)	4.64^a,b^ (0.16)	5.96^a,c^ (0.07)
Hope	2.65^b,c^ (0.26)	4.14^a,b^ (0.16)	4.73^a,c^ (0.08)
Group efficacy	4.21^b,c^ (0.29)	5.00^a,b^ (0.13)	5.78^a,c^ (0.05)
Opinion‐based identification	4.30^b,c^ (0.29)	4.99^a,b^ (0.13)	6.02^a,c^ (0.05)
Collective action intentions	3.26^c^ (0.25)	3.45^a^ (0.13)	4.31^a,c^ (0.08)

*Note*: Different superscripts denote differences between profiles on the outcome variable according to the mixture model: ^a^Denotes that ambivalent future thinkers and change‐oriented utopian thinkers are different (*p* < .05); ^b^Denotes that dystopian thinkers and ambivalent future thinkers are different (*p* < .05); ^c^Denotes that dystopian thinkers and change‐oriented utopian thinkers are different (*p* < .05).

##### Criticism‐related outcomes

Change‐oriented utopian thinkers reported significantly lower system justification than ambivalent future thinkers, X^2^ = 5.70, *p* = .044, and stronger moral convictions about the target issue than the other subgroups, X^2^s ≥ 3.84, all *p*s ≤ .009. Thus, they were generally least satisfied with the status quo compared to the other profiles.

Dystopian thinkers reported similar levels of system justification to the other profiles, X^2^s ≤ 3.75, all *p*s ≥ .089. However, they also reported significantly higher anger than change‐oriented and ambivalent future thinkers, X^2^s ≥ 9.26, all *p*s ≤ .004, whereas the other profiles were not significantly different from each other, X^2^s ≤ 0.32, all *p*s ≥ .573. Dystopian thinkers also reported significantly higher moral convictions about the target issue than ambivalent future thinkers, X^2^ = 7.70, *p* = .002.^2^

##### Change‐related outcomes

As expected, change‐oriented thinkers reported significantly higher opinion‐based identification, hope, group efficacy and collective action intentions than the other subgroups, X^2^s ≥ 8.83, all *p*s ≤ .003. Ambivalent future thinkers also reported higher opinion‐based identification, hope and group efficacy than dystopian thinkers, X^2^s ≥ 6.65, all *p*s ≤ .003, though dystopian and ambivalent future thinkers did not differ on collective action intentions, X^2^s ≤ 1.40, all *p*s ≥ .237.

#### Sensitivity analyses: controlling for study context

To control for any issue‐ and study‐related variation in the profile outcomes, we re‐ran the analyses controlling for the study participants were recruited from. We ran these analyses using the MPlus AUXILARY option with R3STEP as this allowed us to enter covariates in the model (i.e. 3 dummy coded variables with Study 4 (gun control/rights) as the reference category).

The results were identical for anger, hope and collective action. The only changes were: (a) change‐oriented utopian thinkers reported significantly lower system justification than dystopian thinkers, β = −0.55, SE = 0.20, *p* = .006; (b) change‐oriented utopian thinkers still reported significantly higher moral convictions, group efficacy and identification, but the comparisons between dystopian and ambivalent future thinkers became non‐significant, all βs ≤ 0.35, all *p*s ≥ .220; and (c) the difference between ambivalent future thinkers and change‐oriented utopian thinkers on moral convictions became non‐significant, β = 0.40, SE = 0.38, *p* = .297. Thus, although the pattern of effects between dystopian and ambivalent future thinkers changed slightly, change‐oriented utopian thinkers still scored highest on the criticism‐ and change‐related outcomes when controlling for the specific issue/study.

### Discussion

Overall, the results from Phase 1 reveal heterogeneity in people's self‐generated futures, and that *combinations of evaluations* about how desirable, beneficent, innovative and possible their imagined future would be predicted differences in satisfaction with, and intentions to challenge, the current system. However, the results also suggest greater complexity than we anticipated. A subgroup of *change‐oriented utopian thinkers* emerged whose self‐generated futures were high in terms of desirability, beneficence, innovation and possibility. Importantly, change‐oriented utopian thinkers reported significantly higher opinion‐based identification, group efficacy, moral convictions and hope than the other subgroups, and lower system justification. In absolute terms, these were the only people who reported that they intended to take action to change the status quo. This pattern was largely unchanged when including study as a covariate in the model, indicating that these differences were not confounded by the issue participants thought about.

Unexpectedly, the two remaining profiles reflected diverse ways that people construct the future, rather than different types of utopian thinking. We observed a subgroup of *dystopian thinkers* who indicated that their imagined futures were not desirable, beneficial, innovative and neither likely nor unlikely to happen; and a subgroup of *ambivalent future thinkers* who ‘neither agreed nor disagreed’ that their imagined futures were desirable, beneficent or innovative, but saw them as somewhat possible. Both profiles were mixed in their social change support, albeit in different ways. Although dystopian thinkers reported stronger anger than the other profiles, they were lower on hope and collective action intentions. Thus, dystopian thinkers are by no means satisfied with the status quo, yet do not feel hopeful about or inclined to act to effect change (Daysh et al., 2024).

Although ambivalent future thinkers scored higher on some of the change‐related outcomes relative to dystopian thinkers, they also reported stronger system justification and lower collective action intentions (relative to change‐oriented utopian thinkers), and lower anger (relative to dystopian thinkers). Thus, this subgroup reported some degree of change commitment, but were also relatively more satisfied with the current system and less willing to agitate for change.

Though ambivalent future thinkers initially reported lower moral convictions, and higher group efficacy and opinion‐based identification than dystopian thinkers, these differences became non‐significant when controlling for study context. Thus, while some subgroup differences on the criticism‐ and change‐related outcomes were influenced by the specific issue participants were asked to imagine, both profiles still demonstrated mixed social change support relative to change‐oriented utopian thinkers in terms of system critical attitudes (system justification, anger) and a willingness to agitate for change (hope, collective action intentions).

## PHASE 2

Phase 2 sought to clarify the unexpected findings from Phase 1 by further probing the psychology of the three profiles. To do this, we adopted a computerized natural language processing approach using Linguistic Inquiry Wordcount (LIWC; Pennebaker et al., 2015) to analyse people's written descriptions of their self‐generated futures. This approach allowed us to examine the prevalence of certain linguistic markers (described below) that reflect underlying psychological differences between the profiles. Doing so might help to explain how and why these groups construct the future in these unexpected ways, and their varied support for social change.

### Using LIWC to probe psychological differences between the latent profiles

LIWC uses word frequency to yield insight into the meaning of language. Specifically, LIWC uses word‐count methods with pre‐programmed dictionaries that represent semantic categories that correspond to psychological constructs of interest. The LIWC dictionaries have been extensively validated (Pennebaker & Chung, 2007) and triangulated with more conventional forms of measurement to show that language‐based inferences reflect underlying social psychological processes and group differences, including collective action (Smith et al., 2018, 2020), moral values (Day et al., 2014) and group differences in emotions and attitudes (Bliuc et al., 2020). Below, we discuss psychological processes that may explain the unexpected ways the profiles constructed and reacted to future change. We then outline the language sentiments which may evidence these processes and form the basis of our analyses (see Table 5 for a summary of these predictions).

**TABLE 5:** Overview of and associated support for the predictions tested in phase 2.

Hypotheses		Supported
Compared to the other profiles…		
H1	Ambivalent future thinkers' written descriptions will contain a higher proportion of risk‐related language	No
H2	Ambivalent future thinkers' written descriptions will contain a higher proportion of threat‐related language	No
H3	Dystopian thinkers' written descriptions will contain a higher proportion of hopelessness‐related language	Partially
H4	Change‐oriented utopian thinkers' written descriptions will contain a higher proportion of care‐ and fairness‐related language	Partially
H5	Dystopian thinkers' written descriptions will contain a higher proportion of harm‐ and unfairness‐related language	No
H6	Change‐oriented utopian thinkers' written descriptions will contain a higher proportion of words which denote goal‐oriented behaviour	No

#### Ambivalent future thinkers

This subgroup may be engaging in *pragmatic prospection* (Baumeister et al., 2016). When thinking about the future, people identify desirable outcomes *and* consider how to actualize these despite potential obstacles. When asked to imagine a utopia, ambivalent future thinkers may have considered potential setbacks to achieving a desirable future, leading them to hold more tempered or cautious expectations (Oettingen, 2012; Soman & Cheema, 2004) and report lower social change commitment (Doosje et al., 2002; Lizzio‐Wilson et al., 2021, 2022). Carefully weighing risks may be particularly salient in the context of social change, which is often incrementally achieved over the course of multiple setbacks, if at all (Lizzio‐Wilson et al., 2021, 2024; Louis et al., 2020, 2022). If this explanation is correct, ambivalent future thinkers' written descriptions should contain more risk‐related language than the other profiles (H1).

Alternatively, ambivalent future thinkers' responses may reflect *resistance to change*. People can feel threatened by, and react negatively when, status relations in society are perceived as changing, and they are faced with the possibility of losing their privilege (Craig & Richeson, 2014a, 2014b) and/or feel that their group identity is being devalued by drawing attention to group‐based inequalities (Lizzio‐Wilson et al., in press). Although all participants supported the social issue examined in each study, people can notionally support equality while simultaneously worrying about potential threats or losses if parity is achieved (Dworkin et al., 2012; see also Lizzio‐Wilson et al., in press). Thus, ambivalent future thinkers' may have perceived a utopian (equal) future as conflicting with their personal and/or ingroup's status and opportunities, leading them to evaluate this future as less desirable, beneficent and innovative (vs. change‐oriented utopian thinkers), and express greater satisfaction with, and a lower willingness to challenge, the status quo. Ambivalent future thinkers' written descriptions should therefore contain more threat‐related language relative to the other profiles (H2).

#### Dystopian thinkers

Although dystopian thinkers described undesirable futures, it is unlikely that this reflects resistance to change given their stronger anger, suggesting that they are dissatisfied with the status quo. Instead, this profile may perceive the status quo as stable and unlikely to change, resulting in hopelessness (i.e. feeling negatively about the future and capacity to affect change; Aubin et al., 2016). This is consistent with evidence that perceiving inequality as enduring can decrease people's willingness to challenge the status quo (Ellemers, 1993) and lead people to feel hopeless and demotivated about future change (Aubin et al., 2016). Thus, we expect dystopian thinkers' future descriptions will contain more hopelessness‐related language relative to the other profiles (H3).

#### Change‐oriented utopian thinkers

This profile reported the highest investment in change‐related outcomes and, unexpectedly, were generally less satisfied with the status quo than the other profiles. Their imagined futures may thus encompass both the criticism and change‐related functions. If this *dual function* explanation is correct, this might manifest in two ways.

The criticism function may be evidenced by moral language related to care and fairness. Care and fairness are two aspects of moral judgement regarding the protection of individuals and concerns about justice and equity, respectively (Graham et al., 2009). Both are associated with a preference for equality, change and regulation of behaviours that might harm others (Graham et al., 2011). As change‐oriented utopian thinkers were generally more dissatisfied with the status quo relative to the other groups, they may have described future societies that address inequality and promote welfare (i.e. care and fairness).^3^ Thus, the criticism function may be evidenced by more care‐ and fairness‐related language (H4). Conversely, given that dystopian thinkers described ominous futures and reported lower social change support, their written descriptions may contain more harm‐ and unfairness‐related language (H5) reflecting concerns about the continuation of an unjust status quo. Thus, both dystopian and change‐oriented utopian thinkers may use different forms of moral language when describing their futures (compared to ambivalent future thinkers).

If change‐oriented utopian thinkers also use their future visions as a goal to strive towards (per the change function), their descriptions may contain a higher proportion of goal‐oriented language. Although this function can be inferred from their higher collective action intentions, triangulating these results with the language sentiment in their written descriptions would provide further evidence that, for some people, utopias energize societally transformative behaviours (Levitas, 2013) and serve as beacons to ‘build the world they want to live in’ (Rao & Power, 2021). We expect that change‐oriented utopian thinkers' future descriptions will contain more words denoting goal‐oriented behaviour than the other profiles (H6).

### Method

#### Participants

We pre‐registered that we would include all 509 participants from Phase 1. However, 76 participants from Study 3 were subsequently excluded because the study design involved people completing the utopian thinking task individually or as a part of an online group chat with other participants (see Table 1). Although the group chat participants described their imagined futures in the chat logs, the nested nature of these data made it difficult to combine and compare with the individual responses in the other studies. Thus, we only retained participants from Study 3 who completed the utopian thinking individually (*n* = 37), leaving a final sample of 433 (*M*_age_ = 40.47, SD = 12.98, 54.50% men and 45.50% women). Although this is below our pre‐registered sample size and current conventions for LPA and mixture models, recent studies have used sample sizes below this threshold and yielded robust results (Lizzio‐Wilson et al., 2021, 2023).

#### Measures

We used pre‐existing LIWC dictionaries to analyse participants' written descriptions of their self‐generated futures from Phase 1 (see Table 6 for descriptives statistics for each profile). Following Ritter et al. (2014) and Day et al. (2014), we removed duplicate words/word stems across the dictionaries to avoid artificial inflation of word count (see Tables S8–S13 for a comprehensive list). For brevity, Table 7 provides descriptions of each LIWC dictionary and the corresponding psychological constructs they assess, and example future visions captured in each category.

**TABLE 6:** Descriptive statistics for the latent profiles in Phase 2.

	Dystopian thinkers (*n* = 37)	Ambivalent future thinkers (*n* = 109)	Change‐oriented utopian thinkers (*n* = 287)
Number of participants from each study			
Study 1 (economic inequality)	29	18	50
Study 2 (climate change)	6	6	91
Study 3 (climate change)	1	14	22
Study 4 (gun control/rights)	1	71	124
Mean proportion of natural language sentiment (SD)			
Risk	0.13 (0.42)	0.42 (0.98)	0.44 (0.97)
Threat	11.77 (4.77)	14.37 (5.58)	12.72 (5.45)
Hopelessness	0.34 (1.19)	0.11 (0.57)	0.04 (0.21)
Care	0.70 (2.38)	0.30 (0.87)	0.59 (1.35)
Fairness	0.06 (0.28)	0.69 (1.31)	0.41 (0.98)
Harm	0.04 (0.16)	0.14 (0.54)	0.30 (0.93)
Unfairness	0.71 (1.17)	0.16 (0.53)	0.24 (0.70)
Goal‐orientation	1.87 (2.16)	1.19 (1.59)	1.37 (2.07)
Future focus	2.82 (3.95)	2.15 (3.16)	2.60 (3.34)
Mean word count (SD)	86.11 (47.55)	82.70 (39.03)	78.74 (44.85)

*Note*: The descriptive statistics were calculated based on the 433 participants retained in the Phase 2 analyses, not the full sample.

**TABLE 7:** Overview of LIWC dictionaries, corresponding psychological constructs and sample quotations.

Psychological construct	Dictionary name (Reference)	Description	Sample quotations (Participant id) Example words from each dictionary are *italicized*
Risk	LIWC‐22 risk focus dictionary (Pennebaker et al., 2015)	Captures language denoting a preoccupation with concerns, dangers and things to avoid. Higher scores might be indicative of people ‘weighing the risks’ or setting more realistic expectations or goals	We would all be *safer* because everyone would have one gun at least and crime would plummet drastically due to fear of the armed populous…With an armed society we are much *safer* but still need to address the individuals with murderous intent. (P 269) Greater restriction on gun control can eliminate the *dangers* of mass shooting, most cases of homicides, gang wars and other crimes committed. Use of guns only by people who can assured by the government, those who will only opt it at a very hostile situation during self [defence] could bring much peaceful environment in our society. (P 397)
Threat	Grievance dictionary (van der Vegt et al., 2021)	Taps overt violent grievances and, of more relevance to the present study, covert social grievances including frustration and jealousy. The grievance dictionary is suited to capturing language reflecting intergroup threats (see Lanning et al., 2021)	If economic inequality has been reduced, this could mean that there is a *limit* placed on how much someone can *earn*. This may also mean that the minimum wage has been increased. Now having economic equality sounds great actually. It would mean less *jealousy* between groups. (P 35) Well I assume the would be more peaceful as a result. More people would be having kids as people wouldn't *fear* for their child's future in a hopeless *Mad* Max‐style hellscape. If there are wars and what not they would not be over things like potable water or dry land but instead for classical reasons like ideology or conquest or oil. (P 99)
Care	The Moral Foundations Dictionary 2.0 (MFD‐2; Frimer, 2020)	Categorizes moral language into virtue (morally righteous actions) or vice (moral violations) categories	I think society would be more peaceful. I think that people would start to rebuild the economy. I think that people would be more *compassionate* if they had a better quality of life. If you reduce the economic inequality you reduce the chances for crime and violence. People would appreciate things more they would not take things for granted in a equal society. (P17) The oceans are clean again with no trash floating in various depth of the oceans. The fish population has returned and have redeemed their *health benefits* to society. The atmosphere is now cleaner and acid rain has ceased to erode the soils. (P 185)
Harm			Our weather cycles will be less dramatic. The world will be a quieter place. Many of the *ravages* of the past will be corrected or in the process of correction. Human disease will be less. The world will be in much more harmony. (P 169) In a future where there is stricter gun control, I imagine that the crime rate and the number of random shootings would decrease significantly. We would hear far fewer stories of school shootings, armed robberies, and other serious crimes. We wouldn't have to worry about strangers *harming* us out of nowhere. (P 290)
Fairness			In this world, most people can afford to live with emergency savings, and not just ‘paycheck to paycheck’. People can afford healthcare, and people are able to put their kids through school […] people are able to accept each other more because they are not pointing their fingers at the ones who are accepting assistance and claiming they take away hard workers' money. Because people are *compensated* better, they understand when people need a hand out from time to time and not feeling bitter about it. (P 95) People in society would be free to do as they choose, just as the Bill of Rights had intended. They will have the *right* to own and bear arms to hunt, use for sport, and protect their families. They won't have to worry whether or not laws will be discussed to infringe upon those *rights*. (P 405)
Unfairness			A reduction in economic *inequality* should have a direct effect on racial *segregation* in society. This is because differences in wealth are often seen between whites and other races. There would also be a direct effect between inner cities communities and those in the most wealthy suburbs. As those traditionally less wealthy accumulate more money, banks would be freer to lend this money out to directly help the local economy in a rippling effect. There are also certain goods and services traditionally used to help struggling families that would be less in demand since they would be less needed. (P 22) Fossil fuel billionaires and wall street hedge funders and banks were all put in jail for their crimes and the corrupt politicians that they work for were put in jail also […] Then the subsidies and tax cuts that were lost from the *theft* by politicians was used to create solar and wind vehicles and electricity. (P 105)
Goal‐oriented language	LIWC‐22 achievement dictionary (Pennebaker et al., 2015)	Captures language denoting goal‐directed behaviour and mastery of challenging tasks	The state would be a great place to live and *strive* for a *better* future for your family's. You'll be *able* to set realistic *goals* for yourself and your family. You'll be *able* to save money for retirement and be *able* to enjoy retirement. [Poverty] would be low and people would be *able* to get jobs and get off of welfare and housing assistances. (P 71) I think it would be a *better* world, nations would have been *working* together to address the climate change, which would in turn mean less friction between different nations and a greater sense of a global community. (P 224)
Hopelessness	Dejection dictionary (Johnsen et al., 2014)	Captures disappointment at failing to achieve goals and discouragement about achieving these in the future which broadly align with the appraisals preceding hopelessness (intractability and low efficacy; Aubin et al., 2016). We added four additional words/word stems (unlikely, impossible, pointless and what is the point) which might also evidence demotivation and intractability	If economic inequality were reduced in the US it would have a profound effect […] There would be a certain amount of discord, because the top 1 or 2 percent would not *give up* any amount of their wealth easily. Having said all of that, the idea of economic equality is almost overwhelming in the impacts and changes that would have to happen. (P 62) In this world, massive steps will have been taken to reduce dependence on fossil fuels across the board […] Even if by some *impossible* miracle humans did not cause climate change, the effort to reduce our carbon footprint will lead to a better world and a happier population. (P 214)
Future focus	LIWC‐22 future focus dictionary (Pennebaker et al., 2015)	Captures language reflecting future‐oriented cognition	Unemployment will go down, crime *will* go down, and so *will* the poverty line. Also the people will be happier and costs will go down. Over all cost of living should go down as well. (P 61) I think our world *will* be a better place. I think we *will* be leaving a better place for our children and grandchildren. I think we *will* no longer have to worry about sustainability. (P 136)

*Note*: Some example quotations have been edited for length and spellings errors to enhance clarity. Any amendments to text are denoted using square brackets.

### Results

#### Conceptual overlap between the LIWC dictionaries

To assess redundancy between related constructs (e.g. risk and threat; see Smith et al., 2018), we examined the pattern of correlations between the dictionaries while controlling for word count. These analyses revealed that the dictionaries were either unrelated or only weakly associated with one another (see Table S14). Thus, they were tapping distinct constructs (see Data S1).

#### Does natural language sentiment predict profile membership?

The proportion of words in each LIWC dictionary were used to predict profile membership using the MPlus AUXILARY option with R3STEP. These mixture models allowed us to estimate the unique utility of each LIWC category in predicting the profiles analogous to multinomial logistic regression (without treating the profiles as observed variables; Asparouhov & Muthén, 2012). In comparing the predictors of each profile, we used Dystopian Thinkers as the reference group (−1) for Ambivalent Future Thinkers and Change‐Oriented Utopian Thinkers (1), respectively; and Ambivalent Future Thinkers as the reference group (−1) when comparing them with Change‐Oriented Utopian Thinkers (1). The word count of participants' written descriptions was included as a control variable.

Conceptually, it was most appropriate to examine natural language sentiment as a predictor or correlate (rather than an outcome) of profile membership. The study procedure meant that participants first described their imagined future, then provided their self‐report ratings along the four profile indicators. Thus, the language participants used reflects psychological differences in their thoughts about the future which, in turn, shaped the nature and number of the latent profiles.

*Three forms of natural language sentiment* predicted profile membership. Table 5 summarizes which predictions were supported, and Table 8 summarizes the log odds and standard errors for each comparison.

**TABLE 8:** Log odds, standard errors and *p*‐values for predictors of profile membership.

	Dystopian thinkers (−1) vs. ambivalent future thinkers (1)			Dystopian thinkers (−1) vs. change‐oriented utopian thinkers (1)			Ambivalent future thinkers (−1) vs. change‐oriented utopian thinkers (1)		
Natural language sentiment	Log odds	SE	*p*	Log odds	SE	*p*	Log odds	SE	*p*
Risk	0.26	1.03	.804	0.50	0.53	.342	0.25	1.00	.805
Threat	0.04	0.04	.368	0.02	0.04	.655	−0.02	0.03	.455
Hopelessness	−0.04	0.25	.875	**−0.90**	**0.36**	.**013**	**−0.86**	**0.36**	.**017**
Care	0.18	0.30	.549	0.18	0.23	.424	<0.01	0.25	.998
Fairness	**39.76**	**0.13**	**<.001**	**39.70**	**<0.01**	**<.001**	−0.06	0.13	.663
Harm	0.24	1.29	.856	0.69	0.48	.149	0.45	1.27	.722
Unfairness	−0.32	0.23	.172	−0.43	0.23	.061	−0.11	0.18	.524
Goal‐orientation^a^	−0.19	0.11	.077	**−0.17**	**0.08**	.**041**	0.02	0.09	.823
Future focus	0.02	0.09	.845	−0.02	0.06	.768	−0.04	0.07	.605
Word count	−0.01	0.01	.133	−0.01	<0.01	.078	<0.001	<0.01	.956

*Note*: Significant effects are in bold. Word count was included as a covariate.

^a^ The significant effect of goal‐oriented language became non‐significant when controlling for study.

##### Hopelessness

As expected, participants who used more hopelessness‐related language were more likely to be dystopian and ambivalent future thinkers (vs. change‐oriented utopian thinkers). Unexpectedly, dystopian and ambivalent future thinkers used a similar proportion of hopelessness‐related language.

##### Fairness

Consistent with predictions, people who used more fairness‐related language were more likely to be change‐oriented utopian thinkers and ambivalent future thinkers (vs. dystopian thinkers). Unexpectedly, change‐oriented utopian thinkers and ambivalent future thinkers used a similar proportion of fairness‐related language. Care‐, harm‐ and unfairness‐related language did not differentiate the profiles.

##### Goal‐orientation

Contrary to expectations, people who used more goal‐oriented language were *more likely* to be dystopian thinkers (vs. change‐oriented utopian thinkers). There were no other significant differences between the profiles. Consistent with our pre‐registered analyses (and following Smith et al., 2018), the first and fourth authors read and manually coded a subset of participants' written descriptions to clarify these unexpected results. We coded 20% of changed‐oriented utopian thinkers written descriptions. Given the small number of dystopian thinkers, we coded all their written descriptions.

The manual coding revealed that some dystopian thinkers used more goal‐oriented language when describing the futility of striving towards a utopia (e.g. ‘Wealth inequality is set up so that others cannot *get ahead* and others will always be on top’.). Thus, these participants supported reducing inequality but were sceptical that such a future could be achieved. Unexpectedly, other dystopian thinkers did not support reducing inequality because they worried that doing so would negatively impact people's goal‐directed behaviour and willingness to strive for success (e.g. ‘In this world, *incentive* to *succeed* would become non‐existent, because people who *work* harder or smarter will be *rewarded* the same as those who are lazy or uneducated’.). The remaining dystopian thinkers either described the current state of affairs (‘This world has a large gap in income. I feel like the middle class is slowly vanishing and you either make a lot of money or very little money. It is a lot harder for people to survive on the economy we have today’.) or expressed support for fixing an issue without evaluating if these changes were futile (e.g. ‘In my eyes a world with greater gun restriction would lower crime rates. I view the process of buying a gun to be a much more rigorous process’).

In contrast, change‐oriented utopian thinkers described a future where their goals were already achieved rather than something to strive towards (e.g. ‘A world where climate change was ended would also be a world where there'd be less air pollution from fossil fuels, which would also *improve* quality of life for many people, especially disadvantaged communities’).

##### Other natural language sentiment

Risk and threat‐related language did not predict profile membership. We found no differences in the use of future‐focussed language between the profiles. Thus, each group was indeed describing the future during the writing task.

##### Word count

Word count was included in the analyses as a covariate, though the mixture models indicated that word count did not predict profile membership (see Table 8). Thus, each profile wrote roughly the same amount of text, suggesting that, despite their varied social change commitment, they were equally engaged in the writing task.

#### Sensitivity analyses: controlling for study context

As in Phase 1, we re‐ran the analyses controlling for the study participants were drawn from. The results of the covariate and main analyses were almost identical, except that goal‐oriented language no longer differentiated profile membership between dystopian and change‐oriented utopian thinkers, *β* = −0.06, SE = 0.10, *p* = .560. Thus, most differences in language‐natural use between the profiles are connected to different orientations about the future over and above the specific study/issue, while some differences may be context dependent.

### Discussion

Overall, three forms of natural language sentiment differentiated the profiles. Participants who used more fairness‐related language were more likely to be change‐oriented utopian thinkers (vs. dystopian thinkers), suggesting that their imagined futures sought to address inequality and change the status quo.

The results also provided a more nuanced understanding of dystopian thinkers. Participants who used more hopelessness‐related language were more likely to be dystopian thinkers (vs. change‐oriented utopian thinkers), supporting our speculation that this profile feels negatively about their capacity to affect change (Aubin et al., 2016). Unexpectedly, dystopian thinkers reported *higher* goal‐oriented language than change‐oriented utopian thinkers. Manual coding indicated that this reflected two concerns: the futility of striving towards a utopia as a goal, and concerns about equality negatively impacting people's willingness to strive for success. Thus, contrary to our expectations, some dystopian thinkers were reacting negatively to the prospect of change. However, the effect goal‐oriented language became non‐significant when controlling for study. This change does not compromise the validity of the findings, but instead suggests that some aspects of participants' future visions are shaped by the nature of specific social issues (e.g. concerns about addressing inequality), and that some issues may lend themselves more easily to certain forms of future‐oriented thought (we return to this point in the General Discussion).

We found no support for the pragmatic prospection or threat explanations, as all three profiles used similar proportions of risk and threat‐related language. Per the Phase 1 findings, ambivalent future thinkers ‘sat in‐between’ the other profiles regarding their natural language use: they were just as ‘hopeless’ as dystopian thinkers but used a similar proportion of fairness‐related language to change‐oriented utopian thinkers.

## GENERAL DISCUSSION

**FIGURE 2:**
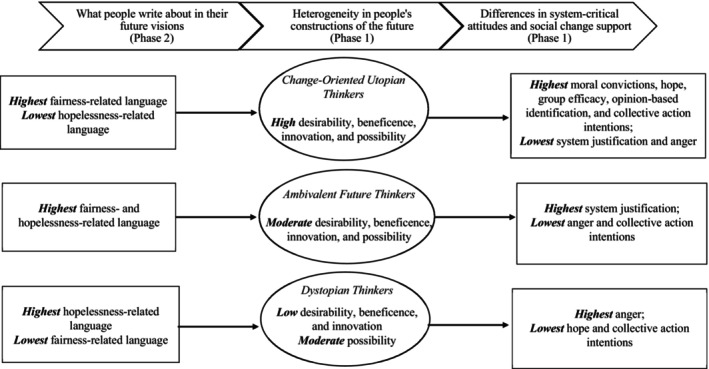
Overview of the final profiles, outcomes and predictors of profile membership across Phase 1 and Phase 2. Circles represent the latent profiles, and squares represent combinations of observed (measured) variables that are expected outcomes of profile membership.

Though previously conceptualized as outcomes of utopian thinking, we examined whether people's self‐generated utopias differ in the extent to which they criticize, seek to change or escape from an undesirable status quo; and if these distinct types of utopian visions predict differences in satisfaction with, and intentions to challenge, the current system. Instead of identifying different types of utopian thinkers, our results suggest that there is heterogeneity in how people construct the future when asked to envisage a utopia. Moreover, the content of these different visions of the future is important in predicting system‐critical attitudes and willingness to agitate for change (see Figure 2 for a summary).

### The features of change‐oriented utopian thinking

As expected, a subgroup of *change‐oriented utopian thinkers* emerged whose imagined futures were high in terms of (self‐reported) desirability, beneficence, innovation and possibility. A computerized linguistic analysis indicated that change‐oriented utopian thinkers used more fairness‐related language, suggesting that the content of their futures centres on promoting justice and equity. Importantly, this profile reported greater dissatisfaction with the status quo (lower system justification, higher moral convictions) and stronger belief in their ability and willingness to challenge the system (higher hope, group efficacy, opinion‐based identification and collective action intentions).

Building on prior work (Fernando et al., 2018, 2020, 2023; Kashima & Fernando, 2020), we examined other features of imagined futures beyond desirability that, in combination, correlated positively with a greater willingness to critique and challenge the status quo. That is, social change commitment was associated with future visions that were not only desirable but also encapsulated pro‐social attitudes that seek to promote welfare and rights (i.e. a social good), innovative alternatives to the status quo to resolve contemporary problems (i.e. how change might happen) and a belief that these changes may come to fruition (i.e. if change can happen). As this was the only profile who engaged in ‘utopian thinking’, we could not compare if different combinations of these features predicted differences in the criticism and change outcomes. We also cannot draw causal conclusions about the effects of these features on the outcome variables because we tested these associations correlationally. However, these findings provide preliminary evidence that there may be other features of utopias which predict their motivational potential.

Although we expected a separate profile of critical utopian thinkers to emerge, change‐oriented utopian thinkers seem to encapsulate both the criticism and change functions. Following Fernando et al.'s (2018) work, this suggests that both functions occur simultaneously, perhaps because feeling dissatisfied with the status quo encourages actions to change ‘what is’ into ‘what could be’ (Kashima & Fernando, 2020; see also Thomas et al., 2012; van Zomeren et al., 2008).

### Not all future visions are motivating: dystopian and ambivalent future thinkers

Two other profiles emerged who, unexpectedly, did not describe utopias: *dystopian thinkers* (whose imagined futures were not desirable, beneficent, innovative and neither likely nor unlikely to happen); and *ambivalent future thinkers* (who ‘neither agreed nor disagreed’ that their imagined futures were desirable, beneficent or innovative, but were somewhat possible). These profiles were generally less critical of, and willing to change, the status quo compared to change‐oriented utopian thinkers. While dystopian thinkers reported stronger anger overall, they were lower on all change‐related outcomes. Ambivalent future thinkers sat in‐between the other profiles on some of the changed‐related outcomes, though they were generally more satisfied with the system and, like dystopian thinkers, uninclined to engage in collective action. Thus, instead of reflecting different types of utopian thinkers, these profiles capture heterogeneity in how people respond when asked to envisage a utopia in and of itself.

A computerized linguistic analysis of dystopian and ambivalent future thinkers' written descriptions helped explain these unexpected future visions. Dystopian thinkers used less fairness‐related language, and more hopelessness‐related and goal‐directed language (vs. change‐oriented utopian thinkers). Thus, dystopian thinkers' ominous outlook was underpinned by two different concerns. For some, this reflected support for addressing inequality coupled with a sense of futility that such change could happen (Aubin et al., 2016; Daysh et al., 2024; Ellemers, 1993). Others, however, were reacting negatively to the prospect of progressive change. Although this was not evidenced by a higher proportion of threat‐related language as we expected, this is likely because participants did not discuss losing their own privilege. Rather, people expressed general concerns that hard work and individual merit would not be rewarded if equality was achieved. These findings suggest that people can abstractly support an issue, but concretely imagining the changes needed to achieve equality might elicit resistance for some. Although these data are correlational, these results may highlight potential backlash effects of utopian thinking.

While ambivalent future thinkers were as ‘hopeless’ as dystopian thinkers, they (and change‐oriented utopian thinkers) used more fairness‐related language. Thus, ambivalent future thinkers may hold tempered expectations about change by imagining a fairer future while simultaneously believing that change is unlikely. This is reminiscent of the anti‐utopianism concept introduced by Fernando et al. (2018) also Levitas (2008), in that ambivalent future thinkers are attempting to be utopian but are constrained by doubts or disbeliefs that such a desirable situation is achievable. Although this is not how we conceptualized an escapist profile, ambivalent future thinkers may cope with their tempered expectations by expressing greater satisfaction with an inescapable system to ‘make the best of a bad situation’ (Kay & Friesen, 2011) and moderate levels of hope as a form of emotion‐focused coping in the face of enduring social problems (van Zomeren et al., 2019). This profile highlights the complicated reactions that people can have when imagining and approaching social change. Although these data are correlational, this finding suggests that imagining ideal future societies may not predict greater social change motivation for all people.

### Considerations for utopian thinking as a method to tackle social problems

By adopting person‐centred analyses, we identified heterogeneity in how people approach imagining ‘utopian’ futures, which, in turn, predict differences in social change commitment. Notably, these effects were obscured when using variable‐centred methods (see Data S1). Although some other studies have found evidence for these effects using variable‐centred analyses (Fernando et al., 2018, 2020), this work asked participants to imagine their own personal vision of an ideal society. In contrast, our approach required imagining and describing the resolution of large‐scale social problems. Though speculative, people may imagine personal utopias in a less heterogeneous way because they feel more control over attaining this vision (Oettingen, 2012) or because they can more easily imagine future change without negative flow‐on effects for themselves or society (Craig & Richeson, 2014a, 2014b). Thus, utopian thinking may have unexpected consequences when used to encourage large‐scale social change commitment. These results may explain why recent experimental work employing this paradigm has not found straightforward effects on social change support (Bird, Thomas, Wenzel, & Lizzio‐Wilson, 2024; Daysh et al., 2024). Accordingly, we propose two considerations for using utopian thinking as a method to address social issues.

First, the context surrounding an issue may influence how people approach the task. Phase 2 indicated that people who used more hopelessness‐related language were more likely to belong to profiles which described dystopian or ambivalent futures, and these profiles, in turn, reported lower hope, group efficacy and action intentions. Although utopian thinking can help groups affirm their efficacy (Badaan et al., 2020, 2022), people who feel that the status quo is enduring (or who are embedded in intergroup contexts characterized by intractable conflict) may be less amenable to these benefits. Thus, future work should explore the contextual factors which impact the effectiveness of utopian thinking, and specifically explore whether overcoming perceptions of intractability affects task engagement.

Second, it may be possible to foster ‘change‐oriented utopian thinking’ by prompting participants to imagine a future which encapsulates the four key features: desirability, beneficence, innovation and possibility. This may prevent people from imagining dystopian and ambivalent futures and have positive flow‐on effects for their social change commitment. However, we cannot draw causal conclusions about the effect of these features based on the current study design. Indeed, people's responses on the profile indicators may reflect (rather than cause) their responses on the criticism‐ and change‐related outcomes. Thus, to determine if these four features are indeed ‘key ingredients’ of utopian thought, experimental research should examine the causal links between future visions which encapsulate these features and relevant outcomes.

### Limitations and future directions

Future research should examine whether some social issues lend themselves more easily to certain types of future‐oriented thinking. While this was not our focus, we re‐ran the analyses controlling for study context to rule out any confounding effects in the Phases 1–2 results. While this largely did not affect the results, the most substantive change was that the difference in goal‐oriented language between dystopian and change‐oriented utopian thinkers became non‐significant. We also conducted separate exploratory mixture models which indicated that the specific issue participants were asked to write about predicted profile membership. Specifically, participants in the economic inequality and climate change studies (Studies 1–3) were generally less likely to be change‐oriented utopian thinkers, and were more likely to be dystopian thinkers or ambivalent future thinkers (see Table S15). In contrast, participants in the gun control/rights study (Study 4) were more likely to be change‐oriented utopian thinkers, and less likely to belong to the dystopian thinkers or ambivalent future thinkers group than participants in the other studies.

These exploratory findings should be interpreted cautiously given that participants in the gun control/rights study were overrepresented in our sample (see Table 1). However, these results provide interesting preliminary evidence that social issues characterized by potential status changes/loss for certain people or groups (economic inequality) or that require large macro‐level changes to avert disaster (climate change) may lend themselves more easily to pessimistic or ambivalent future‐oriented thoughts. Thus, future work should examine whether the nature of the profiles systematically vary across different social issues and/or contexts (e.g. see Yip et al., 2025), and if utopian thinking is more effective in cultivating social change support in relation to different issues.

We identified several natural language sentiments that predicted profile membership. This suggests that some aspects of participants' future visions were different between the profiles, which helped make sense of their unexpected response patterns on the profile indicators. However, the profiles did not differ on the other natural language sentiments as we had predicted (see Table 8 and Figure 2). This may be because the participants in each profile wrote about a similar future world but then rated that same vision differently across the four profile indicators (i.e. in terms of their subjective perceptions of how desirable, beneficent, innovative and possible that vision was). For example, each profile may have used similar care‐related language because they described a future that promoted people's welfare by addressing the specific social issue (e.g. mitigating climate change) but differed in how they appraised that future vision along the profile indicators. This perhaps highlights another nuance in using utopian thinking as a method—people may imagine and describe ostensibly the same thing, but this does not necessarily mean that they will view this vision as positive or report greater social change motivation.

To further explore the connection between the content and motivating potential of imagined futures, research could employ manual qualitative analysis. We adopted a computerized approach to systematically compare natural language sentiment (and their underlying psychological constructs) between the profiles without affecting latent class formation (Asparouhov & Muthén, 2012). However, this approach cannot provide a detailed analysis of language use in context or identify other important features of participants' written descriptions that were not considered a priori. Thus, future work employing traditional qualitative methods can provide a ‘thick account’ of participants' future visions and identify other ‘motivating features’ beyond those considered here.

Finally, comparisons involving dystopian thinkers should be interpreted cautiously given the small number of participants who comprised this group. Although we had adequate power to detect the expected number of profiles, using a larger sample may help determine the stability of the dystopian thinkers profile and allow for more nuanced and reliable analyses.

## CONCLUSION

As illustrated by the opening quote, imagining future possibilities and pursuing social change often go hand‐in‐hand. However, the features of people's future visions have important consequences for their willingness to critique and challenge the status quo. Although we initially sought to identify different types of utopian thinkers, we instead found heterogeneity in how people respond to being asked to describe utopias in and of themselves. Thus, if researchers and change agents want to use utopian thinking as a method to cultivate social change support, they also need to consider how the context surrounding social issues might affect engagement with the task, and how to guide people's approach to the task such that their utopias contain the ‘right ingredients’ for action engagement.

## AUTHOR CONTRIBUTIONS

**Morgana Lizzio‐Wilson:** Writing – original draft; conceptualization; formal analysis; project administration; data curation; supervision; methodology. **Emma F. Thomas:** Funding acquisition; supervision; investigation; conceptualization; writing – review and editing. **Michael Wenzel:** Funding acquisition; conceptualization; writing – review and editing. **Emily Haines:** Writing – review and editing; formal analysis. **Jesse Stevens:** Investigation; methodology. **Daniel Fighera:** Investigation; methodology. **Patrick Williams:** Investigation; methodology. **Samuel Arthurson:** Investigation; methodology. **Danny Osborne:** Funding acquisition; writing – review and editing; conceptualization. **Linda J. Skitka:** Funding acquisition; writing – review and editing; conceptualization.

## FUNDING INFORMATION

This work was supported by an Australian Research Council Discovery Project awarded to the second, third, ninth and tenth authors (DP200101921).

## CONFLICT OF INTEREST STATEMENT

There are no conflicts of interest.

## ETHICS STATEMENT

This research received ethical approval from the first‐eighth authors' institutions and adheres to the APA code of conduct. All participants were briefed about their rights (e.g. right to withdraw, anonymity and potential risks) before providing their informed consent to participate in this research.

## Supporting information


Data S1:

## Data Availability

The survey materials, data files and analysis code are openly available on the Open Science Framework: https://osf.io/8sujz/?view_only=0b60de9553d64fd4912bd7f99d279880. Phase 1 and Phase 2 were both pre‐registered: https://osf.io/cv4re/?view_only=28f94c57c29a4cc2868a79d88656db46 and https://osf.io/6d327/?view_only=2c4ab544497d453aaf90a73b76217eac.

## Footnotes

^1^
In LPA research, ‘outcome’ is used to denote associations between profile membership and variables of interest, rather than cause‐and‐effect relationships (see Osborne & Sibley, 2017; Thomas et al., 2024).

^2^
As moral conviction was not assessed in all studies, we ran separate mixture models on this variable only using the pooled data from Studies 2 to 3.

^3^
Some participants were supporters of conservative causes (more lenient gun laws). While different moral considerations can be salient for liberals and conservatives (Graham et al., 2009), we still expected that care and fairness‐related language would characterize their utopias. This is because supporters of gun rights may describe utopias which address care and fairness concerns regarding gun access (e.g. protecting the right to bear arms, gun access for protection/self‐defence; Stroebe et al., 2017). Thus, both groups may construct futures which address similar moral concerns in relation to their position on gun control (see also Frimer et al., 2013).
