# Supplementary material for: What could be? Depends on who you ask: Using latent profile analysis and natural language processing to identify the different types and content of utopian visions
Source: Br J Soc Psychol. 2025 Feb 3;64(2):e12853. doi: 10.1111/bjso.12853 (PMC11789452; doi:10.1111/bjso.12853)
Supplement: Supplementary file 1 — Data S1: [file BJSO-64-0-s001.docx]

**Variable Centred Analyses: Studies 1-4**

Overall, there were virtually no significant effects observed in Studies 1-4 when using variable-centred analyses (see Tables S1-7). The only significant effects of the utopian thinking task emerged in Studies 1 and 4. In both studies, participants reported stronger anger when thinking about the present versus the future; but also reported greater hope when imagining the future versus thinking about the present (see Tables S1, S6-7).
 Significant main effects of participants’ position on gun control were observed in Study 4, such that gun rights supporters reported higher system justification, group efficacy, opinion-based identification, and hope; while gun controls supporters reported higher collective action intentions and anger (see Tables S6-7).

**Table S1**

*Summary of Cell Means (Standard Deviations) and T-Test Statistics for Study 1 (Economic Inequality)*

| Variable | Future Condition | Present Condition | T-test statistics  (*t*, df, *p* value) |
| --- | --- | --- | --- |
| System justification | 3.29 (1.09) | 3.38 (1.24) | *t*(176) = 0.61, *p* = .541 |
| Anger | 3.45 (1.82) | 4.29  (1.82) | ***t*(176) = 3.00, *p* = .003** |
| Hope | 4.31  (1.90) | 3.49  (1.72) | ***t*(176) = -2.74, *p* = .007** |
| Group efficacy | 4.95  (1.64) | 4.91  (1.75) | *t*(176) = -0.19, *p* = .849 |
| Opinion-based identification | 5.11  (1.67) | 5.04  (1.89) | *t*(176) = -0.35, *p* = .725 |
| Collective action intentions | 3.78  (1.68) | 3.95  (1.60) | *t*(176) = 0.63, *p* = .527 |

*Note.* Significant effects are in bold.

**Table S2.**

|  | ANOVA statistics  (*F*, df, *p* value, η_p_^2^) | | |
| --- | --- | --- | --- |
| Variable | Future Valence | Mental Contrasting | Future Valence x Mental Contrasting |
| System justification | *F*(1, 195) = 0.64, *p* = .425, η_p_^2^ < .01 | *F*(1, 195) = 0.11, *p* = .740, η_p_^2^ < .01 | *F*(1, 195) = 0.40, *p* = .530, η_p_^2^ < .01 |
| Anger | *F*(1, 195) = 0.37, *p* = .543, η_p_^2^ < .01 | *F*(1, 195) = 0.17, *p* = .678, η_p_^2^ < .01 | *F*(1, 195) = 0.55, *p* = .459, η_p_^2^ < .01 |
| Moral conviction | *F*(1, 195) = 1.30, *p* = .265, η_p_^2^ = .01 | *F*(1, 195) = 0.07, *p* = .791, η_p_^2^ < .01 | *F*(1, 195) = 0.07, *p* = .261, η_p_^2^ = .01 |
| Hope | *F*(1, 195) = 1.09, *p* = .299, η_p_^2^ = .01 | *F*(1, 195) = 0.59, *p* = .442, η_p_^2^ < .01 | *F*(1, 195) = 0.07, *p* = .261, η_p_^2^ = .01 |
| Group efficacy | *F*(1, 195) = 1.06, *p* = .304, η_p_^2^ = .01 | *F*(1, 195) = 0.07, *p* = .788, η_p_^2^ < .01 | *F*(1, 195) = 0.97, *p* = .326, η_p_^2^ = .01 |
| Opinion-based identification | *F*(1, 195) = 1.24, *p* = .267, η_p_^2^ = .01 | *F*(1, 195) = 0.23, *p* = .633, η_p_^2^ < .01 | *F*(1, 195) = 0.20, *p* = .655, η_p_^2^ < .01 |
| Collective action intentions | *F*(1, 195) = 0.58, *p* = .446, η_p_^2^ < .01 | *F*(1, 195) = 0.17, *p* = .679, η_p_^2^ < .01 | *F*(1, 195) = 1.30, *p* = .255, η_p_^2^ = .01 |

*Summary of ANOVA Statistics for Study 2 (Climate Change)*

**Table S3**

*Cell Means (Standard Deviations) for the Outcome Variables in Study 2 (Climate Change)*

| Variable | Mental Contrasting Absent | | Mental Contrasting Present | |
| --- | --- | --- | --- | --- |
|  | Negative Future | Positive Future | Negative Future | Positive Future |
| System justification | 3.51 (1.17) | 3.28  (1.24) | 3.35  (1.03) | 3.33  (1.20) |
| Anger | 4.36  (1.84) | 4.68  (1.32) | 4.63  (1.77) | 4.60 (1.62) |
| Moral conviction | 5.41  (1.32) | 5.80  (0.89) | 5.56  (1.40) | 5.56  (1.18) |
| Hope | 3.72  (1.62) | 4.06  (1.66) | 3.62  (1.67) | 3.79  (1.77) |
| Group efficacy | 5.41  (1.61) | 5.80 (1.06) | 5.55 (1.48) | 5.56  (1.28) |
| Opinion-based identification | 5.19  (1.78) | 5.54  (1.41) | 5.40  (1.84) | 5.55  (1.25) |
| Collective action intentions | 4.12  (1.68) | 4.52  (1.36) | 4.27  (1.48) | 4.19  (1.48) |

**Table S4**

*Summary of ANOVA statistics for Study 3 (Climate Change)*

|  | ANOVA statistics  (*F*, df, *p* value, η_p_^2^) | | |
| --- | --- | --- | --- |
| Variable | Time Referent | Group Interaction | Time Referent x Group Interaction |
| System justification | *F*(1, 225) = 0.60, *p* = .815, η_p_^2^ < .01 | *F*(1, 225) = 0.17, *p* = .680, η_p_^2^ < .01 | *F*(1, 195) = 0.83, *p* = .364, η_p_^2^ < .01 |
| Anger | *F*(1, 225) = 0.42, *p* = .507, η_p_^2^ < .01 | *F*(1, 225) = 0.11, *p* = .742, η_p_^2^ < .01 | *F*(1, 195) = 0.84, *p* = .360, η_p_^2^ < .01 |
| Moral conviction | *F*(1, 225) = 0.99, *p* =.321, η_p_^2^ < .01 | *F*(1, 225) = 0.88, *p* =.349, η_p_^2^ < .01 | *F*(1, 195) = 0.69, *p* =.406, η_p_^2^ < .01 |
| Hope | *F*(1, 225) = 0.07, *p* = .786, η_p_^2^ < .01 | *F*(1, 225) = 1.74, *p* = .188, η_p_^2^ = .01 | *F*(1, 195) = 0.21, *p* = .644, η_p_^2^ < .01 |
| Group efficacy | *F*(1, 225) = 0.37, *p* =.546, η_p_^2^ < .01 | *F*(1, 225) = 0.07, *p* =.789, η_p_^2^ < .01 | *F*(1, 195) = 0.03, *p* =.860, η_p_^2^ < .01 |
| Opinion-based identification | *F*(1, 225) <.01, *p* =.955, η_p_^2^ < .01 | *F*(1, 225) = 0.19, *p* =.661, η_p_^2^ < .01 | *F*(1, 195) = 0.38, *p* =.540, η_p_^2^ < .01 |
| Collective action intentions | *F*(1, 225) = 1.44, *p* =.232, η_p_^2^ = .01 | *F*(1, 225) = 0.01, *p* =.940, η_p_^2^ < .01 | *F*(1, 195) = 0.40, *p* =.529, η_p_^2^ < .01 |

**Table S5**

*Cell Means (Standard Deviations) for the Outcome Variables in Study 3 (Climate Change)*

|  | Group Interaction Present | | Group Interaction Absent | |
| --- | --- | --- | --- | --- |
| Variable | Future | Present | Future | Present |
| System justification | 3.59  (1.07) | 3.43  (0.98) | 3.40  (0.97) | 3.50  (0.92) |
| Anger | 4.57  (1.66) | 4.51  (1.57) | 4.27  (1.68) | 4.65  (1.68) |
| Moral conviction | 5.49  (1.16) | 5.51  (1.25) | 5.50  (1.04) | 5.81  (0.85) |
| Hope | 4.61  (1.57) | 4.45  (1.52) | 4.80  (1.19) | 4.84  (1.56) |
| Group efficacy | 5.77  (1.18) | 5.84 (0.98) | 5.78 (0.88) | 5.90  (1.11) |
| Opinion-based identification | 5.86  (1.10) | 5.95 (1.01) | 5.89 (1.05) | 5.78  (1.35) |
| Collective action intentions | 4.52  (1.35) | 4.63  (1.09) | 4.42  (1.32) | 4.76  (1.51) |

**Table S6**

*Summary of ANOVA Statistics for Study 4 (Gun Control)*

*Note.* Significant effects are in bold.

|  | ANOVA statistics  (*F*, df, *p* value, η_p_^2^) | | |
| --- | --- | --- | --- |
| Variable | Time Referent | Position on Gun Control | Time Referent x Position |
| System justification | *F*(1, 391) = 1.37, *p* = .242, η_p_^2^ < .01 | ***F*(1, 391) = 16.39, *p* < .001, η_p_^2^ = .04** | *F*(1, 391) = 1.15, *p* = .285, η_p_^2^ < .01 |
| Anger | ***F*(1, 391) = 23.29, *p* < .001, η_p_^2^ = .06** | ***F*(1, 391) = 19.07, *p* < .001, η_p_^2^ = .05** | *F*(1, 391) = 3.67, *p* = .056, η_p_^2^ = .01 |
| Hope | ***F*(1, 391) = 21.53, *p* < .001, η_p_^2^ = .05** | ***F*(1, 391) = 6.12, *p* = .014, η_p_^2^ = .02** | *F*(1, 391) = 2.95, *p* = .086, η_p_^2^ = .01 |
| Group efficacy | *F*(1, 391) = 1.47, *p* = .227, η_p_^2^ < .01 | ***F*(1, 391) = 12.71, *p* < .001, η_p_^2^ = .03** | *F*(1, 391) = 0.17, *p* = .683, η_p_^2^ < .01 |
| Opinion-based identification | *F*(1, 391) = 3.56, *p* = .060, η_p_^2^ = .01 | ***F*(1, 391) = 9.05, *p* = .003, η_p_^2^ = .02** | *F*(1, 391) = 3.79, *p* = .052, η_p_^2^ = .01 |
| Collective action intentions | *F*(1, 391) = 0.08, *p* = .776, η_p_^2^ < .01 | ***F*(1, 391) = 5.79, *p* = .017, η_p_^2^ = .02** | *F*(1, 391) = 0.01, *p* = .925, η_p_^2^ < .01 |

**Table S7**

*Cell Means (Standard Deviations) for the Outcome Variables in Study 4 (Gun Control)*

|  | Gun Rights Supporters | | Gun Control Supporters | |
| --- | --- | --- | --- | --- |
| Variable | Future | Present | Future | Present |
| System justification | 4.07  (0.99) | 3.86 (0.96) | 3.58  (0.93) | 3.57  (0.92) |
| Anger | 2.42  (1.37) | 2.90 (1.69) | 2.83  (1.62) | 3.93  (1.82) |
| Hope | 4.81  (1.53) | 4.33  (1.52) | 4.68  (1.58) | 3.65  (1.82) |
| Group efficacy | 5.70  (0.98) | 5.79 (0.98) | 5.26  (1.19) | 5.44  (1.26) |
| Opinion-based identification | 6.13  (0.99) | 6.12  (0.88) | 5.65  (1.08) | 6.02  (0.89) |
| Collective action intentions | 3.56  (1.65) | 3.59  (1.69) | 3.93  (1.40) | 3.99  (1.52) |

**Revised LIWC Dictionaries**

The revised LIWC dictionary files used in our analyses can be found on the project OSF page: <https://osf.io/8sujz/?view_only=0b60de9553d64fd4912bd7f99d279880>

**Table S8**

*Revised LIWC-22 Achievement Dictionary*

- All duplicated words (33 of 277) were removed (highlighted in yellow)
- Words in achievement dictionary which signify failure/failing to achieve goals (31 of 277, highlighted in green) were moved to the Dejection Dictionary

| abilit* | able | accomplish* | ace | aced | aces | achiev* | acing | actualiz* | advantag* | aim | aimed | aiming | aims |
| --- | --- | --- | --- | --- | --- | --- | --- | --- | --- | --- | --- | --- | --- |
| ambition* | ambitious | ambitious* | aspiration* | aspire* | aspiring | attain | attainabl* | attained | attaining | attainment | attains | award* | best |
| better | burnout* | capab* | capable | celebrat* | celebrate | certif* | certified | challeng* | challenging | champ | champion* | champs | climb* |
| climbed | commitment | committed | compet* | competitive | confidence | confident | confidently | conquer* | conscientious* | contend* | create | created | creates |
| creating | creation* | creativ* | dedicat* | determina* | determinedly | diligen* | dropout* | earn | earned | earns | effective* | efficien* | effort |
| eminen* | endeav* | excel | excelled | excellent | excelling | excels | exceptional* | expert* | fail* | fall* behind | fame | famous | famous* |
| fell behind | finalist* | finish* | flunk* | founded | founder* | founding | ftw | fulfill* | fulfilling | gain* | gave up | generativ* | get* ahead |
| give* up | glories | glorifi* | glorify* | glory | go getter* | go-getter* | goal | goal-orient* | goal-setting | goals | gold medal* | gpa | grad |
| grads | graduat* | grandmaster* | hard work* | hard-work* | hardwork* | honor* | honour* | improve* | improving | inabilit* | incapab* | incentive* | incompeten* |
| ineffect* | initiative* | invent* | irresponsib* | lazier | laziest | laziness | lazy | lead | leading | leads | lose | loser* | loses |
| losing | mastered | masterful* | mastering | masters | mastery | mba | medal* | mediocr* | merit* | meritocrac* | motivat* | multitask* | opportun* |
| outstanding* | overcame | overcome | overcomes | overcoming | overconfiden* | overtak* | peerless* | perfected | perfecting | perfection | perfectly | perfects | persever* |
| persist* | phd* | plan | planned | planner | planning | plans | post graduate | post-graduate | practice | practiced | practicing | prais* | pride |
| prize | procrastin* | productiv* | productive | proficien* | progress | promot* | promotion | proud | prouder | proudest | proudly | pull* ahead | qualifi* |
| qualify | quit | quits | quitt* | ranked | ranking* | reap | reaped | reaping | reaps | record break* | record-break* | regain* | renown* |
| resourceful* | reward* | rewarding | sacrific* | skill* | solution* | solve | solved | solves | solving | strateg* | strategize* | striv* | succeed* |
| succeeded | success | successes | successful | successfully | super star* | super-star* | superb | superbly | superstar* | surpass* | talented | tip-top | tiptop |
| tournament* | tried | tries | triumph* | triumphant* | triumphantly | trophies | trophy | try | trying | unable | unbeat* | unmotivated | unproduc* |
| unqualified | unsuccessful* | victor | victories | victorious* | victors | victory | visionar* | win | winner | winner* | winners | winning | winnings |
| wins | won | work | workahol* | worked | working | works | world class | world-class | efficient | practices |  |  |  |

**Table S9**

*Revised LIWC-22 Risk Focus Dictionary*

- All duplicated words (30 of 128) were removed (highlighted in yellow)

| advisories | advisory | alarmed | alarming* | alert* | apprehens* | averse | aversion | aversive | aversively | aversiveness | avert* | avoid* | avoidant | avoided |
| --- | --- | --- | --- | --- | --- | --- | --- | --- | --- | --- | --- | --- | --- | --- |
| avoids | balk | balked | balking | balks | careful | caution | cautioned | cautious | consequen* | conservative* | crises | crisis | danger | dangerous |
| dangerously | dangers | dare | dared | daring | daringly | daunt* | daunting | dauntless | distrust* | escap* | evacuat* | evad* | feasib* | feel* expos* |
| fled | flee | fleeing | flees | forbod* | gambl* | harm | harmful | hazard | hazarding | hazardous | hazards | heedless* | hesita* | hid |
| hidden | hide | hides | hiding | insurance | liabil* | liable | life threatening | life-threatening | looming | lose | loses | losing | loss | lotter* |
| luck | lucked | luckier | luckiest | luckily | lucky | mess* around | misfortune | pain | pained | painful | painful* | pains | peril* | pessimis* |
| pessimistic | prevent* | preventative care | preventative medicine | protect* | protective | reckless | reckless* | reluctan* | risk* | safe | safest | safety | save* | saving* |
| scrupulous* | secur* | threat* | trouble | troubles | troublesome | tw | unprotected | unsafe | vigilance | vigilant | vigilantly | waried | waries | warily |
| wariness | warn | warned | warning* | warns | wary | evas* | safer |  |  |  |  |  |  |  |

**Table S10**

*Revised LIWC-22 Future Focus Dictionary*

- All duplicated words (18 of 138) were removed (highlighted in yellow)

| 2nite | about to | ahead | anticipate* | anticipation | approaching | attainabl* | destined | destiny | eventual | eventually | expect* | fate | fated | fates |
| --- | --- | --- | --- | --- | --- | --- | --- | --- | --- | --- | --- | --- | --- | --- |
| feasib* | feasible | finna | fixin | fixin’ | fixin* to | flashforward | forbod* | foresaw | foresee* | foreshadow* | foresight | forseeabl* | forthcoming* | futur* |
| going | going to | gon | gonna | gotta | gunna | has to | has to be | have got to | have to | he’ll | heading | henceforth | hereafter | hope |
| hoped | hopeful* | hopefully | hopes | hoping | how’ll | howll | i’ll | i’mma | ill | ilyk | ima | imma | imminen* | impending |
| it’ll | itll | l8r | looming | make sure | may | might | might’nt | mightn’t | obtainable | oncoming | onward | plan | planned | planner |
| planning | plans | posterity | postpon* | potential | potentially | pray | prayed | prayer* | praying | prays | predict* | prepar* | prepare* to | promising |
| propos* | prospect* | ready to | shall | shan’t | shant | she’ll | someday | sometime | soon | sooner | soonest | supposed to | tba | tbd |
| that’ll | thatll | thereafter | they’ll | theyll | tmrw | tmw | to-morrow | tomorrow* | ttyl | up and coming* | up-and-coming* | upcoming | wanna | wants |
| we’ll | what’ll | whatll | where’ll | wherell | who’ll | wholl | why’ll | whyll | will | will do | wish | wishes | wishing | won’t |
| wont | you’ll | youll |  |  |  |  |  |  |  |  |  |  |  |  |

**Table S11**

*Revised Grievance Dictionary*

- All duplicated words (252 of 2453) were removed (highlighted in yellow). We removed words related to the future (e.g., future, future goal and aim) such that these only appears in the future focus dictionary.
- An extra 5 words were deleted as the same word appeared twice in the dictionary (highlighted in blue).

| 911 | 155mm | abandon* | abduct* | abhor* | abid* by | abus* | accid* | accompan* | accomplic* | accomplish* | accurac* | accus* | achiev* | acrid |
| --- | --- | --- | --- | --- | --- | --- | --- | --- | --- | --- | --- | --- | --- | --- |
| act* as | act* of terror | act* upon | action* | activ* | actor* | admir* | adopt* | adulter* | advers* | adversar* | advis* | advisor* | adwar* | aerial* |
| affair | affection* | affil* | affright* | afghan* | afraid | aftermath | agenda* | agendum* | aggrav* | aggress* | aggressor* | aggriev* | agit* | agon* |
| aid* | aim* | air forc* | air forc* offic* | air out | aircraft* | airfield* | airforc* | airmen | ak-47 | al-qur’an | alarm* | alarum* | alcohol* | alert* |
| alien* | all-encompass* | all-might* | all-pow* | allah | alleg* | allegi* | alli* | almight* | alon* | alterc* | altogeth* | ambul* | ambush* | amelior* |
| amend* | american* | ammo | ammunit* | amor | amour | analysi* | analyz* | ancient | angel* | anger* | anglican* | angr* | angst | anguish |
| animos* | annoy* | answer* | antagonist* | antenna* | anti-aircraft* | anti-semit* | anti-soci* | antiaircraft | antitank | anxiet* | anxious | apart | apocalyps* | aplogis* |
| apostl* | apostol* | appreci* | apprehend* | approach* | archbishop* | architect* | architectur* plan* | argu* | argument* | arm* | arm* combat | arm* forc* | arm* person* | arm* servic* |
| armageddon | armament | arm* | arm* intellig* | arm* tank* | armor* | armour* | armour* combat vehicl* | arous* | arrang* | arrest* | arrog* | arson | articl* | artiller* |
| ask* | asphyxi* | assail* | assassin* | assault* | assess* | assign* | assist* | associ* | atom* | atomic-war* | atroc* | attach* | attack* | attack aircraft* |
| attempt* | attend* | attend* to | attent* | audio* | aunt* | auschwitz | author* | automat* face recognit* | avaric* | aw | awar* | award* | ax | axe* |
| backbreak* | backup* | bad | badger* | baghdad | ballist | bang* | bankruptc* | baptist* | barbar* | bash* | battalion* | battl* | battle-field* | battler* |
| battleship* | bazooka* | be* after* | be* intim* | be* watch* | bear wit* | beaten | beau* | befor* | beg* | beggar* | begrudg* | behavior* | behaivour* | belief* |
| believ* | belong* | belt* | belt* down | bemoan* | benefici* | benefit* | beret* | betray* | better half | bewilder* | bibl* | biblic* | big brother | bigotr* |
| biolog* | biowarfar* | bipolar | birthday | bisexu* | bitch* | bite* | bitter | black-market | blackbal* | blade | blast* | bleed* | bless* | blood |
| blood* | bloodsh* | bloodthirst* | blow* up | bludgeon* | blue | bluehydra* | blueprint* | bod* | bogus | bomb* | bomber | boom | booster* | boredom |
| boss* | brother* | bottl* up | boundar* | boyfriend | brainsick* | brass knuck* | brave | braver* | brawl* | break down | break-up | breakup* | bride | bring down |
| broke* | brother | brother* love | brows* histor* | bruis* | brutal* | brute | brutish | buddhism | build | build up | built | bullet* | bully* | bullshit |
| bullyrag | bump off | bureau | burglar* | buri* | burn* | busy* | businesslike | cache* | cadet | calendar | caliber | call* | call* for | call* out |
| callos* | camrader* | camcord* | camera* | camo* | camouflag* | camp | campaign* | cannon* | captur* | cardiologist | care | caress | caretak* | carr* into action |
| carr* out | case | casualt* | catastroph* | cathedr* | cathol* | catholic | caught | cause of death | cctv | ceas* | ceasefire | celebr* | cellphone* | cellular telephone |
| cemetery | center of attention | center* on | central intelligence agency | ceremoni* | chain-of-command | chanc* | chao* | chapel | chaplain | charg* | charit* | chat | chat up | cheat |
| cheater | check* | check* up on | cherish* | chevi* | chief | childhood | children | chip | choke* | christ | christendom | christian | christian bible* | christian church* |
| christian religion | chronolog* | church* | church build* | church servic* | churchman | cia | civil* partnership* | clandestine | clash* | class | clemenc* | clench* fist* | clerg* | cleric* |
| clinical depression | clock | clock time | close* | close* off | close-fit | closed-circuit | co | co-ordin* | coaction | cogit* | col | collabor* | collis* | colonel |
| combat | combat injur* | combat-read | come on | come togeth* | command | command* offic* | commando* | commemor* | commit | committ | committed | communic* | community service* | companion |
| companionship | compass | compat* | complain* | complaint | complet* | complex | complic* | compon* | compose* | compuls* | comput* | comrade | comradeship | con |
| con-artist | con-man | conceal | concentr* | concentr* on | concept | concern | conclud* | conclus* | condemn* | condom* | confess* | confin* | conflict | confront |
| congratul* | congreg* | conman | connect | connector | conquer* | conscienc* | consecr* | consequ* | consid* | consider* | conspire* | conspirac* | constabular* | consult |
| consum* | contact | contemn* | contempl* | context | conting* | contraband | control* | controvers* | convers* | convict* | convinc* | cooper* | coordin* | Cop |
| copul* | corp | corps | corros* | corrupt* | counterfeit* | counterintellig* | counterterror* | countr* | couple* | coupl* up | courag* | court | courteous | cousin |
| cover | covert | covet | craze* | crazi* | creat* | creation | creator | credibl* | creep* | cry | cry for help | cr* out | crime | crime of passion |
| crimefight* | crimin* | criminal offenc* | criminal offens* | criminal prosecut* | criminal record* | criminalis* | crisi* | croak* | crown of thorn* | crucify | crucifixion | cruel | cruelty | crusade* |
| cuddle* | cult | cun* | current* | curriculum | cut | cyanide | cyberbull* | d.p.r.k. | dagger | damag* | danger* | dark* | dart | data |
| data collect* | database* | date | date stamp | daughter* | day of the month | dead | dead bod* | deaden | dearest | death | decapit* | deceas* | deceit* | decenc* |
| decept* | decide* | decis* | decompos* | dedic* | defeat* | defence | defence department* | defence force* | defence team* | defend* | defense lawyer* | deit* | deliber* | delirium |
| delud* | delus* | delusion | demand | dement* | demis* | demolish* | department of defence | depend* | depersonalise disord* | depersonalise* neurosi* | deplor* | deploy* | depress* | depression disorder* |
| depriv* | deput* | design* | desir* | desol* | despair | desper* | despic* | despise* | destitute | destroy | destruct* | detach* | detail* | detect |
| detect* work* | detector* | determin* | deterr* | detest* | deton* | detriment* | devast* | develop | device* | devise* | devot* | devoted | die* | differ* |
| differ* stage | difficult* | difficulti* | dignif* | dignit* | diocesan* | dire | disadvantag* | disappoint* | disastr* | discharg* | disciplin* | discomfort | disconcert* | disconnect* |
| discont* | discov* | discrimin* | discriminator* | discuss* | disdain | disench* | disengag* | disfavour | disgrac* | disgruntl* | disguis* | disgust* | dishonest* | dishonor* |
| dislik* | dismay | dismemb* | disord* | disori* | dispatch* | displeas* | displeasur* | dispos* | disput* | disrupt* | dissatisfact* | dissens* | dissert* | disson* |
| distast* | distraught | distress* | distrust* | disturb* | divin* | divorc* | dna | do work | do-or-die | document* | dodg* | doesn’t belong | done | doubt* |
| downcast | dprk | drama* | dread | dream* | drive* forc* | driven | drop* dead | due | due date | duet | duo | dupe* | duper* | duplic* |
| dut* | eager* | ear* | earpiec* | eavesdrop* | ecclesiast* | econom* aid | eden | effect* | effort* | ego* | egoist* | elder* | electrocute* | elysian* |
| embitt* | embrac* | emerg* | emerg* brake | emot* | empathet* | employ* | empt* | emt | enabl* | end* | end goal | end is in sight | end of tether | endang* |
| endanger | endear* | endors* | enem* | enforc* | engag* | engin* | engross | enhanc* | enmit* | ennobl* | enquir* | enrag* | ensur* | entertain* |
| entit* | entranc* | entrust* | envy | envious | environ* | equal* | equip* | erot* love | escal* | espi | espionage | essay | esteem | etern* |
| ethic* | ethic* code | eubstanc* | evacu* | evangel* | evas* | evid* | evil | exact | examin* | exasper* | excel | excit* | excruc* | execr* |
| execut* | exercise | exhaust | expect* | expert | explod* | explos* | expose | exposur* | extend | extension | extrem* | extremist | eye* | eyewit* |
| fabl* | face | face recognit* | facial recognit* | facilit* | failur* | fair | faith* | fake | faker | fallac* | fals* | falsehood | falsif* | famil* |
| family line* | family unit | familiar | fanat* | farsighted | fascin* | fatal* | father | fatigu* | faux | fbi | fear* | fearsom* | fed up | federal agency |
| feel | felicit* | fellowship | felo-de-s* | felon* | felt | feroc* | fianc* | fierc* | fight* | fight* back | fight* down | fighter | figur* out | final |
| final exam | final examin* | final stage* | financ* | financial aid | financial backing | financial support | find | find out | fingerprint* | finish* | finish | finish up | fire | fire arm* |
| fire brigade* | firearm* | firemen | firepower | fishing | fixat* | flamethrow* | fleet | flirt* | flood | focal point | focus* | focus* on | focuss* | foe |
| foeman | follow | fond | footage | force | foreign mission | forensic* | forensic science* | foresight | foresighted | forethought | forever and a day | forfeitur* | forgiv* | forgiving |
| forgotten | forlorn | form | forthcom* | fought | foul | fraca* | fragil* | framework* | frantic | fraud | fraudster | fraudul* | friend | friendly relationship |
| friendship | fright | frighten | frighten away | frighten off | frustrat* | fsb | full general | fun | function | fund | funer* | fury | furious | future* |
| future goal and aim | gabriel | gallant | ganesha | gang* | gas | gay | gay woman | gaze | gchq | gear up | general | genocide | geoint | get it on |
| get laid | ghast* | girlfriend | give | give* up | give-up the ghost | glare* | glbt | gloomy | glory | glum | go ballistic | go out | go steady | goal |
| god | god almighty | god willing | goddam | goddamn | goddess | godless | godlike | good | good shepherd | good will | gore | gossip | govern | govern activ* |
| govern* agenc* | grab | grace | grandchildren | grandfather | grandmother | grandparent* | gratitude | grave | great depression | great power | greatest | greed | greedy | grenade* |
| grief | grievance* | grievous | grim | grisly | groom | ground force* | groupy | grudge | gruesome | guantanamo | guard | guard duty | guerrilla-warfare | guilt |
| guilt feel* | guilty | guilty conscience | gun | gun for hire | gunfire | gunman | gunmen | gunshot | gunsling | habit | hack | hacker | hades | hallucin* |
| handgun* | hang* | happy | harass | hard time | hardship | hariri* | harm* | harass* | harsh | hassle | haste | hasten | hatchet | hate |
| hate crime* | hatred | have intercourse | have sex | hazard | head game | head trip | headset | heart | heart and soul | heart-to-heart | heartache | heartbreak | heaven | heavy weapon |
| helicopt* | hell | help | helper | helpless | helpline | helpmate | herculean | hermit | hero | heroism | hidden | hierarch* | higher be* | hilt |
| hire gun | hit | hit man | hitman | hoax | hoy polloy | hold | hold back | hold open | holy | holy book | holy man | holy person | holy place | holy scripture* |
| holy terror | hold word | holy writ* | holiday | holler | hollo | homeless | homework | homicid* | homophobia | homosexual | hon | honest | honesty | honor |
| honour | hopeless | hornswoggl* | horrible | horrific | horror | hospital* | hospitalis | hostage* | hostil* | howitz* | hubby | hug* | human* | human be* |
| human relationship | humanitarian | hung | hunger | hurry | hurt | husband | hush-hush | hysteria | icbm | idea | ident* element | identif* | ideology | idolatry |
| idolis | ie | ignor* | ill | illeg* | illicit | illog* | illus* | imag* | imitat* | immedi* | immin* | immor* | immort* | impass |
| impati* | impending | imperil* | imperson* | implement* | important | impost* | impot* | imprison* | improvise* | improvised explosive device* | incapacit* | incarcer* | incid* | incite* |
| individual | inexperienced person | infantry | infatuation | infidelity | infiltrat* | inflame | inflict | influenc* | info | inform | infract* | infrastructure | infuriate | inhuman |
| inhuman treatment | injure* | injured party | injury | injustice | inmate | innocent people | inquire | inquiry | insan* | insecur* | insensit* | inspect* | instabl* | instruct* |
| instruction execution | instrument | insuffici* | integrity | intelligence | intellignece activity | intelligence agency | intelligence information | intelligence operat* | intelligence service* | intend | intent* | interloper | intern* | interven* |
| intima* | intimacy | intimid* | intoler* | intricacy | intrude | intrusion | invasion | invect* | invest* | investigation | invincible | invisible | involved | ip-bas* |
| ira | irak | iraq | irat* | ire | irk | irrationale | irregular | irrevers* | irrevoc* | irrit* | isol* | israel-palestine | israel/palestine | jab |
| jail | jealous | jealousy | jehovah | jeopardis | jesus | jesus christ | jesus of nazereth | jittery | job | joined | joint | joy* | jrotc | judaism |
| judgement | judgment | jump* | jump off | jump out | justice | justice department | kabul | keep* | keep an eye on | keep company | keep in line | keylogger | kgb | kibosh |
| kick* | kid | kidnap | kidnapp* | kill | killer | kin | kind | kinfolk | kinsfolk | kinsperson | kiss | knife | knife thrust | knight |
| knives | knuckle duster | koran | kuwait | lady friend | lapd | lash out | last chance | last choice | last hope | last resort | lasting | launcher | law | law-break |
| lawsuit | lay out | leadership | learn | leatherneck | leave | leave behind | legacy | legal action | lesbian | level-headed | lgbt | lgbti | lgbtq* | liar |
| lie | lie in wait | lieuten* | life | life history | life-threaten* | life-time | lifeless | lifetime | ligature | like | limit* | limit point | line of reason | link |
| linkup | listen* | loath* | loathsome | logistic* | loiter* | loner | lonely | lonesome | long | long-run | long-term | longer-term | longterm | longtime |
| look | look after | look at | look into | look on | look out | look over shoulder | lookout | loom* | looting | lord | lose | lose hope | loss | lost |
| love | love affair | love life | love story | lovemak* | lover | loving | loving-kind | low | loyal | loyalist | loyalty | lt. | luftwaff* | lugubri* |
| lunat* | lust | m-16 | macabre | machete | machine gun | machine-gun | machinegun | mad | maim* | maj. | major power | make | make believe | make love |
| make out | make-believe | maker | makingup | malaria | malef* | malefactor | malevolent | malice | malicious | malware | man and wife | manageable | mangl* | manic |
| manic depression | manic depression illness | manic-depression psychosis | manipulate | manslaughter | manslayer | map | march* | marine* | marit* | maritime | married | married couple | married man | married person |
| married woman | marriage | marriage ceremony | masjid | masking | masquerade | mass | massacr* | mastermind | match | matchete | matchless | mate | material | matrimony |
| maturity date | mayday | mayhem | measure | medal | medallion | medic | medication | meet | mele | melior* | memory | menace | mental | mental disease |
| mental disorder | mentally disturbed | mental health | mental illness | mentat* | merciless | message | messiah | metaljacket | method* | mi5 | mi6 | microphone | mif* | mighty |
| milestone | milit* | military | military action | military capable | military control | military force* | military group | military machine | military mission | military officer* | military posture | military service | military strength | military unit |
| militia | militiamen | mind | mind-map | mindless | miser | misery | misgiven | mishandle | misidentify | misled | mislead | misogyni | misrepresent | miss |
| missile | mission | missionary work | mistreat | mistress | mistrust | mixed-up | mobile phone | model | molest | monastery | monitor | monitoring device | monogam* | monogamy |
| monster | monument | mood | moral | moral principle* | morbid | morose | mortar | moslem* | mosque | mother | motivate | mourn family and friend | mourner | muhammad |
| mulan | munition* | murder | must | mutilate* | mutual | mystery | mystery story | myth | mythology | napalm | narcissist | narcotic | nasty | national |
| national security agency | natural depression | naval | naval force* | navy | nco | near | need | needy | neediest | negative | negative charge | negative emotion | negative | neglect |
| nemesis | nerve* | nervous | network | neurologist | neurotic* | neutralis* target | newspaper | newspaper publisher | newsworthy | next | nexus | nice | no escape | no friend* |
| no way out | nobility | noble | nobleman | noblemen | nobody | nominat* | nonexistent | nongreagari* | nonsocial | north korea | not appreicate* | Not qualified | not suppose* to be here | note |
| notice* | nsa | nuclear | nuisance | nuptials | nurse* | nurture | nutcase | nypd | o.d. | obedi* | oblig* | obliter* | observ* | obsess* |
| obsession | obsessive-compulsi* | ocd | offence | offend | offensive | office | old | older | omnipotent | on edge | one | onslaught | open fire | open up |
| opensource intelligence | operational phase | opponent | oppose | opposite | oppress | oppressor | optimist | oracle | orchestrate | order | order of business | ordnance | organise | organised religion |
| orotund* | orphan* | ostracise* | out-of-control | outburst | outcast | outcome | outcry | outline | outrage | overanalyze | overbearing | overdose* | overdue | overhear* |
| overjealous | overreaction | overview | overwhelming | oxfam | padre | pain | pain in the ass | pain in the neck | painful sensation | pair off | pakistan | panic | panic attack | panick |
| pantheism | paper | parade | paradise | paramedic | paramilitary | paranoia | paranoid | parent | parish | particular date | partner | partnership | pass* away | passion |
| pastor | patriot | patrol | patronage | pattern | pauper | peace | pecking order | pediatrician | pedophile | peeve | pentagon | people | peril | perish |
| permanent | perpetrator | perpetual | person* | personnel | personnel casualty | perturb* | petite | pharo | philosophical system | philosophy | phobia | phobic disorder | phone | phoney |
| phony | photographic camera | phratri* | physician | physician-assist* | picture* | piece of work | pierce | pilgrimage | pill | pissed | pissed off | pistol | plan | plan of attack |
| planner | playactor | plea | plead | please | pledge | plot | plutonium | poison* | poisonous substance | police | police detection | police force | policeman | policemen |
| polite | polite sympathy | polygyn* | polyth* | poor | portfolio | portray | possess | possibility | post-traumatic | posttraumatic | posttraumatic stress disorder | poverty-stricken | power | powerless |
| practice | praise | pray | prayer | pre-defined | Pre-determined | preach | preacher | precaution | precious | precise | predefine | predetermine | prejudice | preoccupation |
| prepared | prepared | pressure | pressure level* | pressure sensitive | prestige | pretend | prevent | previsit | prey | pride | priest | principles | priority | prioritise |
| prison | prison house | private | probe | problem | process | procrastinat* | proctor* | profession | program | programm | progress | project | projectile | promise |
| promised land | promote | prophecy | prophesise | prophet | propose | prosecute | prospici* | protect | protest | protestant | proud | provide | provoke | psychiatry |
| psychiatrist | psychological | psychological discord | psychologist | psychopath | psychosis | psychotic | psychotic belief | public | public service | pull the trigger | pulverise | punch | puncher | punish |
| purdah* | purpose | pursual | push | put down | put to death | putin | qaeda | qaida | quarrel | quick | quran | rabbin | race murder | racial |
| racial discrimination | racism | racist | radar | radicalis* | raf | rage | raid | rampage | rape | rapid | rapist | rapture | rash decision | razor blade |
| reach | reach out | read | ready | recapture | reckless | recluse | recoil | reconnaissance | reconnaissance mission | record | record book | rector | red | red alert |
| red army faction | red-faced | reenforce | regret | regular army | regulatory | reinforce | reject | relate | relationship | rely | reliable | reliance | relief | relieve |
| religious | religious belief | religion | remarry | remarriage | remember | remembrance | remorse | remote | renown | repel | report | reprehensive | reputation | request |
| require | rescue | rescuer | resent | reserve | resign | resilient | resources | respect | respond | response | restless | resurrect | retaliate | retaliatory |
| revenge | reverent* | revolt | revulsion | ricin | rifle | right thing to do | righteous | rigid | rigid mind-set | riot | rip | risk | risk of exposure | risk of infection |
| rites | ritual | roadmap | robbery | rocket | rocket-propelled | roil | role | role player | roleplay | romance | romantic | roommate | rootkit | rope |
| rotc | rotten | route | royal air force | rule | run a risk | rush | russia | russian soviet federated socialist republic | s.o.s. | sacred | scared value | sacrosanct | sad | saddam |
| safeguard | safety | safety device | saint | same-sex | samurai | sanctify | savager* | savior | saviour | scam | scar | scare | scare away | scare off |
| scarey | scary | scath | scene | schedule | scheme | schizoid | schizophrenic | schizophrenic disorder | schizophrenic psychosis | schizophrenia | schizotype | schizotype person | school | school day |
| schoolwork | scientific | scorn | scourge | scout | scramble | scream | screen | scud | scuffle | search | searchable | secluse | secrecy | secret |
| security | security department | security measure | security system | see | see red | seen | segregat* | self harm | self-denial | self-destruct | self-doubt | self-harm | self-injury | self-loath* |
| self-mutilate | self-pride | self-worth | selfish | semiautomatic | send away | send off | senseless | sensor | sentence | sentry | sentry duty | separate | sergeant | serial |
| serious | sermon | serve | service | set | set out | set up | set-apart | sever | sex | sexual activity | sexual | sexual love | sexual practice | sexual urge* |
| shady | shaky | sham | shame | shammer | share |  | shed blood | shipboard soldier | shiva | shock | shoot | shoot down | shooter | short fuse |
| shot | shout | shout out | show | showdown | shrapnel | shriek | shrine | sibling | sicken | sight | signal detect | silence | sin | sincerest |
| single | sir | sketch | skil | skill | slash | slay | slayer | sleep together | sleep with | sleuth | slice | slue | slyness | small-arm |
| smash | smasher | smooch | smother | snap | snappish | sneaky | snipe | snog | snoop | snuggle | sociable | social network | society | sociopath |
| soldier | sole | solid | solitary | solitary confinement | solitude | solitudinarian | solo | solve | somber | son | sorry | sorrow | sos | soul |
| soviet russia | spear | spy | spirit | spiritual | spite | spot | spotter | spouse | spousal relationship | spywars | squadron | stab | stable | staff |
| stage | stalemate | stalk | stalker | stare | start | starve | state | state of war | station | steel | step on it | stepfather | stock | stop |
| stop point | straighten out | strain | strand | strange | stranger | strangle | strangul* | strategy | strength | strengthen | stress | strict | stricter | strike |
| stringent | strong | struck | structure | struggle | student | study | stung | success | suffer | suffocate | suicide | suicide terror | suicide vest | superior general |
| supernatural | superpower | supervise | supervisor | supplier | support | suppress | surgeon | surrender | surround | surveil | survey | survive | survivor | suspect |
| suspense | suspicious | suspicion | swindler | sword | synagogue | syria | system | tabs | tactic | take | take down | take into custody | take life | take note |
| taliban | talibanis* | talk | tank | tank car | tantrum | tape | tardili* | target | target area | task | taunt | team | team up | teamwork |
| teamwork | tear | teardrop | tech | technology | telecom | television camera | temper | temperate | temple | tend | tendent* | tense | tense up | tension |
| terminate | termination point | terminus ad quem | terrible | terrified | territory | terror | terrorism | terrorist | terrorist act | terrorist attack | testament | the nazaren | theism | theist |
| theolog* | thieve | think | think ahead | thorough | thought | thought process | threat | threaten | throne | throw in the towel | time | time consume | time limit | time manage |
| time pressure | time run out | time to come | timeframe | timeline | tire of | to-do | together | torah | torment | torpedo | torture | touch | trace | track |
| tradition | traffick | tragedy | tragic | train | traitor | transgression | trauma | traumatic | traumatise | treachery | treasure | try anything | try everything | trying time |
| trial | trick | trickery | trickster | trigger-happy | triggerman | troop | trouble | trueheart | trueness | truncheon | trust | trusting | trustworthy | truth |
| turbulence | turret | tv | tv camera | twin | twosome | tyranny | u.s. air force | u. s. army | u.s. | ufo | ugly | unacceptable | unaccompany | unappreciate |
| unblink | uncle | unconnected | unconscioncess | uncontrol | uncover | underappreciate | undercover | undercover agent | undercover work | understand | undertaken | unease | uneasy | unfair |
| unhappy | unhealthy | unhinge | unicef | uniform | union | union of soviet socialist republic | united | united state air force | united state army | united states department of defense | united states navy | united states of america | unity | universe |
| unjust | unknown | unlike | unloved | unmask | unpredictable | unquestioned | unreal | unrelated | unrest | unrestrained | unruly | unsafe | unsatisfied | unseen |
| unstable | untrust | untrusted | untrusty | untrustworthy | untruth | unwell | unwelcome | unwilling | upcoming | uphold value | upset | uptight | uranium | urgent |
| us air force | us army | us navy | usa | usaf | usurp | valentine | valiant | value-system | value-system | vandal | vanquish | vaticin | vehem | vehement |
| vendetta | venerate | vengeance | venom | vent | verdict | veteran | veteran soldier | vex | vexated | vicar | vicious | victim | video | video record* |
| videotape | viet nam | vietnam | vietnam war | view | vile | villain | violate | violence | violent | violent death | violent disorder | vishnu | vitriol | vitup* |
| vow | vpn | vulnerable | walk away | walk off | want | war | war machine | warfare | warhead | wary | warm | warmhearted | warmth | warn |
| warning device | warning signal | warplane | warrior | warship | wartime | watch | watch out | watch over | watchdog | weapon | weapon system | weaponry | wedding | wedding ceremony |
| wedding party | wedlock | weekend | weep | weird | welfare | well being | well-informed | well-organised | well-thought-of | whodunit | whole | wick | wicked | widow |
| wife | wig | wipe out | wipeout | wiretap | wish | wit | without | woe | wonder | word of god | word of honor | work | work on | work out |
| work toward | workplace | world war | worry | worse | worship | worshipp | worthless | wound | wrath | wretch | wrong | wrongdoer | ww1 | ww2 |
| wwi | wwii | xenophobia | yearn | yell | yob | zeus | doom* |  |  |  |  |  |  |  |

**Table S12**

*Revised Dejection Dictionary*

- All duplicated words (16 of 56) were removed (highlighted in yellow)
- 31 words from Achievement Dictionary denoting failure/failing to achieve goals were added (from burnout* onwards).

| unhapp* | frustrat* | irrit* | deject* | demotivat* | heartsick | depriv* | stoop* | sad | negativ* | sorrow | miss | depress* | miser* | tragic |
| --- | --- | --- | --- | --- | --- | --- | --- | --- | --- | --- | --- | --- | --- | --- |
| sorry | hopeless | despair | mean | bitter | unlikely | impossibl* | pointless | what* the point | burnout* | dropout* | fail* | fall* behind | fell behind | flunk* |
| gave up | give* up | inabilit* | incapab* | incompeten* | ineffect* | irresponsib* | lazier | laziest | laziness | lazy | lose | loser* | loses | losing |
| mediocr* | procrastin* | quit | quits | quitt* | unable | unbeat* | unmotivated | unproduc* | unqualified | unsuccessful* |  |  |  |  |

**Table S13**

*Revised Moral Foundations Dictionary*

- All duplicated words (207 of 2034) were removed (highlighted in yellow)

| abhor | abhored | abhors | abstinance | abstinence | abused | abuser | abusers | abuses | abusing | ache | ached | aches | aching | achingly |
| --- | --- | --- | --- | --- | --- | --- | --- | --- | --- | --- | --- | --- | --- | --- |
| acquiesce | acquiesced | acquiescent | acquiesces | acquiescing | addict | addicted | addicting | addiction | addictions | addicts | adhere | adhered | adherence | adheres |
| adhering | admiral | admirals | adulterer | adulterers | adultery | afflict | afflicted | affliction | afflictions | afflicts | against us | agonize | agonized | agonizing |
| agonizingly | agony | alcoholic | alcoholics | alcoholism | all for one | allah | allegiance | allegiances | allegiant | alleviate | alleviated | alleviates | alleviating | alleviation |
| allied | allies | ally | almighty | altruism | altruist | am partial | anarchist | anarchistic | anarchists | anarchy | angel | anguish | anguished | anguishes |
| anguishing | annihilate | annihilated | annihilates | annihilation | annihilator | apostate | apostates | apostle | apostles | arrest | arrested | arresting | arrests | assassin |
| assassinate | assassinated | assassinates | assassinating | assassination | assassinations | assassins | assault | assaulted | assaulter | assaulters | assaulting | assaults | Atone | atonement |
| atones | atoning | attack | attacked | attacker | attackers | attacks | authorities | authority | authorize | authorized | authorizer | authorizes | authorizing | avenge |
| avenged | avengers | avenges | avenging | backstab | backstabbed | backstabber | backstabbers | backstabbing | backstabs | bamboozle | bamboozled | bamboozles | bamboozling | barf |
| barfed | barfing | barfs | be partial | beatification | beatify | beatifying | bedlam | been objective | been partial | befoul | befouled | befouling | befouls | behind their back |
| behind their backs | being objective | being partial | belong | belonged | belonging | belongs | beneficence | beneficiary | benefit | benefits | benefitted | benefitting | benevolence | benevolent |
| betray | betrayal | betrayed | betrayer | betrayers | betraying | betrays | bias | biased | biases | biasing | bible | bibles | biblical | bigot |
| bigoted | bigots | bilk | bilked | bilking | bilks | bishop | blackmail | blackmailed | blackmailer | blackmailers | blackmailing | blackmails | bless | blessed |
| blesses | blessing | blessings | blood | bloodiness | bloody | bm | body | boss | bossed | bosses | bossing | bow before | bow down | bowing |
| brothers in arms | brutalisation | brutalise | brutalises | brutality | brutalization | brutalize | brutalized | brutalizes | brutalizing | buddha | buddhas | buddhist | bullied | bullies |
| bully | bullyboy | bullying | bullys | by the book | captain | captained | captains | care | cared | caregiver | cares | caring | caringly | carnage |
| caste | castes | casualties | casualty | cathedral | catholicism | catholics | celibacy | celibate | ceo | chaos | chaotic | chaotically | charitable | charity |
| chaste | chastity | chauvinists | cheat | cheat on | cheated | cheated on | cheater | cheating | cheating on | cheats | cheats on | chief | chiefs | chieftain |
| child | childbearing | childbirth | childcare | childhood | christ | christian | christians | church | churches | civil right | civil rights | clan | clans | clean |
| cleaned | cleaner | cleaners | cleaning | cleanliness | cleans | clique | cliques | clothe | coalition | coalitions | cock | cockroach | cockroaches | cocksucker |
| cocksuckers | coerce | coerced | coerces | coercing | coercion | cohort | cohorts | coldhearted | collective | collectively | collectives | comfort | comforted | comforting |
| command | commandant | commanded | commander | commanders | commanding | commandingly | commandment | commandments | commands | commune | communes | communion | communities | community |
| companies | companion | companions | companionship | companionships | company | compassion | compassionate | compassionately | compensate | compensated | compensates | compensating | compensation | compliance |
| compliant | compliantly | comply | comrade | comradery | comrades | con artist | con artists | condolence | condolences | congregate | congregates | congregating | congregation | connive |
| connived | conniver | connives | conniving | consecrate | consecrated | consecrates | consecrating | consecration | console | consoled | consoles | consoling | consolingly | contagion |
| contagious | contagiously | contaminant | contaminants | contaminate | contaminated | contaminates | contaminating | contamination | control | controlled | controller | controllers | controlling | controls |
| corporate ladder | corporate ladders | corps | corpse | corpses | corrupt | corrupting | corruption | corrupts | countries | country | covenant | covenants | crappy | cried |
| crier | cries | crook | crooked | crooks | cruel | cruelness | cruelty | crying | cult | cults | cum | cunt | cunts | curse |
| cursed | curses | cursing | damage | damaged | damager | damages | damaging | damn | damnation | damned | damning | damns | dean | death do us part |
| debase | debased | debases | debasing | debaucherous | debauchery | decay | decayed | decaying | decays | deceit | deceive | deceived | deceivers | deceives |
| deceiving | deceivingly | decency | deception | decree | decreeing | decrees | deference | deferential | deferentially | defile | defiled | defiler | defilers | defiles |
| defiling | deflower | deflowered | deflowering | deform | deformed | deforming | deformities | deformity | deforms | defraud | defrauded | defrauder | defrauding | defrauds |
| degradation | degrade | degraded | degraders | degrades | degrading | degradingly | deification | deify | deism | deist | deists | deities | deity | deprave |
| depraved | depravities | depravity | desecrate | desecrated | desecrates | desecrating | desecration | desecrations | deserter | deserters | deserting | desertion | destroy | destroyed |
| destroyer | destroyers | destroying | destroys | destruction | deviant | deviants | devil | devout | dictate | dictated | dictates | dictating | dictation | dictator |
| dictators | did rob | die | dignified | dignifies | dignify | dignifying | dignities | dignity | dirt | dirtied | dirties | dirty | dirtying | disadvantaged |
| disarray | disarrayed | discomfort | discomforted | discomforting | discomforts | discriminate | discriminated | discriminates | discriminating | discrimination | disease | diseased | diseases | disgust |
| disgusted | disgusting | disgustingly | disgusts | dishonest | dishonesty | dishonor | dishonorable | dishonored | dishonoring | dishonors | dishonouring | dishonours | disloyal | disobedience |
| disobedient | disobey | disorder | disordered | disordering | disparity | disproportion | disproportionate | disproportionately | disproportioned | disproportions | disrespect | disrespected | disrespecting | disrespects |
| dissent | dissented | dissenter | dissenters | dissenting | dissention | dissents | dissident | dissidents | distress | distressed | distresses | distressing | distressingly | distrust |
| distrusted | distrustful | distrusting | distrusts | divine | divine right | divinities | divinity | divorce | divorced | divorces | divorcing | do as one says | do unto others | dominant |
| dominate | dominated | dominates | dominating | dominion | dominions | double cross | double crossed | double crosser | double crossers | double crosses | double crossing | douchebag | douchebags | drug |
| drugged | drugging | drugs | due process | due processes | due processing | dues | dung | dupe | dupes | duping | duplicitous | duress | duties | dutiful |
| duty | economic disparity | egalitarian | egalitarians | elder | elderly | elders | elevating | empathised | empathises | empathising | empathize | empathized | empathizers | empathizing |
| empathy | emperor | emperor | empire | empires | endanger | endangered | endangering | endangers | enemies | enemy | enlist | enlisted | enlisting | enlistment |
| enlists | enshrine | enshrined | enshrines | enshrining | epidemic | epidemics | equal | equalities | equality | equalize | equalizer | equalizers | equals | equitable |
| equities | equity | eternal | fellows | eternally | exalt | exalted | exalting | exalts | excrement | excrete | excreted | excretes | excreting | exploit |
| exploitation | exploited | exploiter | exploiters | exploiting | exploits | exterminate | exterminated | exterminates | exterminating | extermination | exterminator | exterminators | eye for an eye | faction |
| factions | fair | fairminded | fairness | fairplay | faith | faithful | faithfully | faiths | false advertise | false advertised | false advertisement | false advertiser | false advertisers | false advertises |
| false advertising | false impression | false impressions | false witness | familiar | familiarities | familiarity | families | family | fatalities | fatality | father | fathered | fathering | fathers |
| favoritism | fecal | feces | feed | feeder | feeding | feeds | fellow | fellowship | fellowships | fester | festered | festering | festers | fidelity |
| fight | fighter | fighters | fighting | fights | filial piety | filth | filthy | fleeced | fleecing | flesh | follower | followers | food | foods |
| forbade | forbid | forbidding | forbiddingly | forbids | fornicate | fornicated | fornicating | fornication | fornicator | fornicators | foul | fraud | frauds | fraudulent |
| free rider | free riders | freeload | freeloader | freeloaders | freeloading | fuck | fucked | fucker | fucking | fucks | gangrene | gangrenous | garbage | generosity |
| generous | generously | genocidal | genocide | genocides | gentleness | germ | germs | glories | glorious | gloriously | glory | go back on | god | goddess |
| godesses | godless | godliness | godly | gods | golden rule | gonorrhea | gospel | gospels | govern | governed | governess | governing | governor | governors |
| governs | gross | grossness | group | grouped | grouping | groups | guide | guides | guiding | hallow | hallowed | hallowing | hallows | harass |
| harassed | harasser | harassers | harasses | harassing | harassment | harlot | harlots | harm | harmed | harmful | harmfulness | harming | harms | harsh |
| harsher | harshness | hazing | hazings | head honcho | heal | healed | healer | healers | healing | heals | health | healthcare | healthier | healthiness |
| healthy | heaven | heavenly | heavens | hedonism | hedonist | hedonistic | hedonists | hell | help | helped | helper | helpers | helpful | helpfulness |
| helping | helps | herd | herded | herder | herders | herding | herds | heresies | heresy | heretic | heretical | heretics | hierarchical | hierarchies |
| hierarchy | power | holiness | holy | holy cross | holy crosses | homeland | homelands | honest | honesty | honor | honorable | honored | honoring | honors |
| hoodwink | hoodwinked | hoodwinking | hoodwinks | hooker | hookers | horde | hordes | horrific | horrifying | horror | horrors | hospitable | hospital | hospitalise |
| hospitalises | hospitalising | hospitality | hospitalization | hospitalize | hospitalized | hospitalizes | hospitalizing | hug | hugged | hugger | hugging | hugs | humane | hunger |
| hungered | hungering | hungers | hurt | hurtful | hurting | hurts | hypocrisy | hypocrite | hypocrites | illegal | illegality | illegals | imam | imams |
| imbalance | imbalanced | imbalances | immaculate | immaculately | immune | immunities | immunity | impartial | impartiality | impiety | impious | impolite | imposter | imposters |
| impure | impurities | impurity | in charge | incest | incestuous | incestuously | indecencies | indecency | indecent | indecently | indivisible | inequalities | inequality | inequities |
| inequity | infect | infected | infecting | infection | infections | infectiousness | infects | infest | infestation | infested | infesting | infests | infidel | infidelity |
| infidels | inflict | inflicted | inflicting | inflicts | ingroup | inhospitable | inhospitality | inhuman | inhumanity | injured | injurer | injurers | injures | injuring |
| injurious | injury | injustice | injustices | insider | insiders | institution | institutional | institutions | insubordinate | insurrection | insurrectional | insurrectionist | insurrections | integrity |
| jesus | joining | justice | justices | justification | justified | justifies | justify | justifying | justness | karma | kill | killed | killer | killing |
| kills | kin | kindness | kinship | koran | koranic | law | lawful | lawfully | lawless | lawlessness | laws | lawyer | lawyered | lawyering |
| lawyers | lead by example | leader | leaders | leadership | leper | lepers | leprosy | level playing field | level playing fields | level the playing field | level the playing fields | lewd | lewdness | liar |
| liars | lice | lied | lionize | lionizes | lionizing | lord | lorded over | lords | love | loved | lover | loves | loving | lovingly |
| loyal | loyalties | loyalty | lying | malevolence | malevolent | maltreat | maltreated | maltreating | maltreatment | manager | managerial | managers | manhandle | manhandles |
| manure | mar | marred | marriage | married | marries | marring | marry | marrying | mary | master | matriarch | matriarchal | matriarchs | matriarchy |
| mentor | mentored | mentoring | mentors | merciful | mercy | mislead | misleader | misleaders | misleading | misleadingly | misleads | misrule | misruled | misruling |
| mistreat | mistreated | mistreating | mistreatment | mistreats | modesty | moldy | molest | molestation | molested | molester | molesters | molesting | molests | mommy |
| monarch | monarchical | monarchies | monarchs | monarchy | monasteries | monastery | monastic | monasticism | monk | monks | mooch | mooched | moocher | moochers |
| mooches | mooching | mosque | mosques | mother | mothered | motherhood | mothering | motherly | mothers | muck | mucking | mucky | mud | murder |
| murdered | murderer | murderers | murderess | murdering | murderous | murders | mutinied | mutinies | mutinous | mutiny | nation | nations | nausea | nauseated |
| nauseating | nauseatingly | nauseous | necrophilia | necrophiliac | necrophiliacs | needier | neediness | needy | nobility | noble | nobles | nonconforming | nonconformist | nonconformists |
| nonconformity | nun | nunneries | nunnery | nuns | nurse | nursed | nursemaid | nursery | nurses | nursing | nurturance | nurture | nurtured | nurturer |
| nurturers | nurtures | nurturing | obedience | obedient | obediently | obey | obeyed | obeying | obeys | objectiveness | obscene | obscenity | old glory | oligarch |
| oligarchies | oligarchs | oligarchy | one for all | oppress | oppression | order | ordered | ordering | orders | organic | organically | organics | organization | organizations |
| orthodox | orthodoxies | orthodoxy | outgroup | outgroups | outsider | outsiders | overpower | overpowered | overpowering | overpowers | overthrew | overthrow | overthrowing | overthrown |
| overthrows | pain | pained | painfulness | paining | pains | pandemic | pandemics | pandemonium | parasite | parasites | parasitic | parasitically | parenting | parity |
| partiality | pastor | pathogen | pathogenic | pathogens | patient | patients | patriarch | patriarchs | patriarchy | patriot | patriots | pay back | payback | pecking order |
| permission | permissive | permissiveness | permit | permits | persecute | persecuted | persecutes | persecuting | persecution | perverse | perversely | pervert | perverted | perverts |
| pestilence | phlegm | phlegmatic | phlegmatically | pickpocket | pickpocketed | pickpocketing | pickpockets | piety | pigsty | pious | pitied | pities | pity | pitying |
| pityingly | plagiarism | plagiaristic | plague | plagued | plagues | plaguing | player | pledge | pledged | pledger | pledgers | pledges | pledging | police |
| policing | polite | politeness | pope | pray | prayed | prayer | prayers | praying | prays | prejudice | prejudiced | prejudicing | preside over | president |
| presidential | presidents | priest | priestly | priests | prime minister | prime ministerial | prime ministers | principal | principals | pristine | profane | profanities | profanity | promiscuity |
| promiscuous | proper | prophet | prophetic | prophetically | prophets | proportional | proportionality | prostitute | prostituted | prostitutes | prostituting | prostitution | protect | protected |
| protecter | protecters | protecting | protection | protective | protectiveness | protector | protectorate | protectors | protects | prude | puke | puked | pukes | puking |
| punch | punched | puncher | punchers | punches | punching | punish | punished | punisher | punishers | punishes | punishing | punishingly | punishment | punishments |
| punitive | pure | pureness | purification | purified | purifier | purifiers | purifies | purify | purifying | purities | purity | pus | putrid | queen |
| queens | rabbi | rabbinical | rabbis | rabble rouser | rabble rousers | rabble rousing | racism | racist | racists | raise hell | raises hell | raising hell | rank | ranked |
| ranking | ranks | rape | raped | rapes | raping | rapist | rat | rats | ravage | ravaged | ravages | ravaging | raw | rebel |
| rebelled | rebelling | rebellion | rebellions | rebels | reciprocal | reciprocity | referee | refereed | refereeing | referees | refine | refined | refinement | refines |
| refining | refuse | refused | refuser | refusers | refuses | refusing | regulation | regulations | relief | relieve | relieved | reliever | relievers | relieves |
| relieving | religion | religions | religiosity | religious | religiously | renegade | renegades | repaid | reparation | reparations | repay | repaying | repayment | repayments |
| repays | repent | repented | repenting | repents | repugnance | repugnant | repugnantly | repulsed | repulses | repulsing | repulsive | repulsiveness | rescue | rescued |
| rescuer | rescuers | rescues | rescuing | respect | respected | respectful | respectfully | respecting | respects | restitution | retaliate | retaliated | retaliates | retaliating |
| retaliation | retribution | retributions | revenge | revenger | revere | revered | reverence | reverend | reverential | reveres | revolting | righteous | righteously | righteousness |
| rights | ringleader | ringleaders | riot | rioted | rioter | rioters | rioting | riots | ripoff | ripoffs | ripped off | ripping off | rips off | risque |
| robbed | robber | robbers | robbing | robs | rock the boat | rot | rotten | rottenness | rubbish | ruling | sacred | sacredness | sacrifice | sacrificed |
| sacrifices | sacrificial | sacrificing | sacrosanct | safe | safeguard | safekeeping | safely | safeness | safety | saint | saintliness | saintly | saints | sanctified |
| sanctifies | sanctify | sanctifying | sanctity | satan | satanic | satanically | scabies | scam | scammed | scammer | scammers | scamming | scams | scatalogical |
| scripture | scriptures | scum | scummy | scuzz | scuzzy | sect | sects | sedition | segregate | segregated | segregates | segregating | segregation | servant |
| servants | sexism | sexist | sexists | sexual | sexuality | share | shared | sharer | shares | sharing | shat | shit | shite | shits |
| shitter | shittier | shitting | shitty | shrine | shyster | shysters | sin | sinful | sinfully | sinfulness | sinned | sinner | sinners | sinning |
| sins | skank | skanked | skanking | skanks | skanky | slave | slaved | slaves | slaving | slavishly | sleaze | sleaziness | sleazy | slime |
| slimy | slut | sluts | sluttiness | slutting | slutty | smite | smother | smothered | smothering | smothers | social order | sodomy | soiled | solace |
| solidarities | solidarity | sorrow | sorrowful | sorrows | soul | soulful | souls | spiritual | spirituality | spoil | spoiled | spoiling | sportsmanship | spotless |
| spotlessly | spotlessness | spreading | square deal | square dealer | square dealers | square dealing | square deals | stabbed | stabber | stabbers | stabbing | stabs | stacked deck | stacked the deck |
| stacking the deck | stacks the deck | stain | stained | staining | stainless | stains | stature | steal | stealing | steals | sterile | sterility | stole | stolen |
| submission | submissions | submit | submits | submitted | submitter | submitters | submitting | subordinate | subordinated | subordinates | subordinating | subordination | subversion | subvert |
| subverted | subverting | subverts | suckered | suffer | suffered | sufferer | suffering | suffers | sullied | sullies | sully | supernatural | supervise | supervised |
| superviser | supervisers | supervises | supervising | supervision | swear | swearing | swears | swindle | swindled | swindler | swindlers | swindles | swindling | swore |
| sympathize | sympathizer | sympathizers | sympathizing | sympathy | synagogue | synagogues | tabernacle | taint | tainted | tainting | taints | take orders | take up arms | taking advantage |
| talmudic | tarnish | tarnished | tarnishes | tarnishing | tarnishment | team player | temperance | temple | temples | theft | theological | theology | thief | thieves |
| thieving | threat | threaten | threatened | threatening | threateningly | threatens | threats | tit for tat | toe the line | together | top dog | top gun | torah | torahs |
| torment | tormented | tormenter | tormenters | tormenting | tormentor | torments | torture | tortured | torturer | torturers | tortures | torturing | torturous | tradition |
| traditional | traditions | traitor | traitors | transgress | transgressed | transgresses | transgressing | transgression | trash | trashed | trashiness | trashing | trashy | treacherous |
| treachery | treason | tribal | tribalism | tribe | tribes | tribulation | tribulations | tribunal | tribunals | tricked | tricking | trickster | troop | trooper |
| troopers | troops | trouble maker | troupe | troupes | trust | trusted | trusting | trusts | trustworthiness | trustworthy | tumult | tumultuous | unadulterated | unauthorized |
| unbias | unbiased | uncaring | uncaringly | uncharitable | unchaste | unclean | uncleanliness | uncompassionate | undefiled | underling | underlings | undivided | unequal | unequaled |
| unfair | unfairness | unfaithful | unfaithfulness | ungenerous | unharmed | unharmful | unhelpful | unhelpfulness | union jack | unite | united | uniter | uniters | unites |
| uniting | unity | unjust | unkind | unkindness | unlawful | unlawfully | unlawfulness | unmerciful | unpatriotic | unprejudiced | unruly | unspoiled | unsullied | untainted |
| untouchable | untouchables | untouched | untrustworthiness | untrustworthy | uprising | uprisings | us against them | venerate | venerated | venerates | venerating | veneration | vengeance | vermin |
| vice president | victim | victimises | victimization | victimize | victimizer | victimizers | victimizes | victimizing | victims | violence | violent | viral | virgin | virginal |
| virginity | virgins | virus | viruses | vomit | vomits | vomitted | vomitting | vulnerability | vulnerable | war | warmhearted | was partial | waste | wasted |
| waster | wasters | wastes | wasting | wear the crown | wholesome | wholesomely | wholesomeness | whore | whored | whorehouse | whorehouses | whores | whoring | wife |
| will rob | will share | willing | worship | worships | wound | wounded | wounding | wounds | yogi | yogis | atoned | avenger | bowed | captaining |
| cathedrals | catholic | connivers | corrupted | deceiver | defrauders | fuckers | killers | sufferers |  |  |  |  |  |  |

**Table S14**

*Correlations Between LIWC Dictionaries and the Proportion (Standard Deviation) of Each Natural Language Sentiment in Participants’ Written Descriptions*

| LIWC Dictionary | Risk | Goal-orientation | Future | Hopelessness | Care | Harm | Fairness | Unfairness | Threat |
| --- | --- | --- | --- | --- | --- | --- | --- | --- | --- |
| Risk | - |  |  |  |  |  |  |  |  |
| Goal-orientation | <-.01 | - |  |  |  |  |  |  |  |
| Future focus | -.05 | -.05 | - |  |  |  |  |  |  |
| Hopelessness | -.04 | .10* | -.08 | - |  |  |  |  |  |
| Care | .05 | .03 | .03 | -.05 | - |  |  |  |  |
| Harm | .28*** | -.07 | .12* | -.03 | -.03 | - |  |  |  |
| Fairness | -.02 | -.08 | -.04 | -.02 | -.06 | -.08 | - |  |  |
| Unfairness | -.07 | .09 | -.09 | .07 | .03 | -.07 | -.07 | - |  |
| Threat | .19*** | -.15** | .04 | -.10* | <-.01 | .05 | .14** | -.08 | - |
| Proportion  (Standard deviation) | 0.41  (0.94) | 1.37  (1.97) | 2.51  (3.35) | 0.11  (0.53) | 0.53  (1.38) | 0.25 (0.84) | 0.45  (1.05) | 0.26  (0.73) | 13.53  (5.51) |

*Note*. **p* < .05. ***p* < .01. ****p* < .001. Word count was controlled for in these analyses.

**Table S15**

|  | Ambivalent Future Thinkers (-1)  versus  Dystopian Thinkers (1) | | | Ambivalent Future Thinkers (-1) versus  Change-Oriented Utopian Thinkers (1) | | | Dystopian Thinkers (-1) versus  Change-Oriented Utopian Thinkers (1) | | |
| --- | --- | --- | --- | --- | --- | --- | --- | --- | --- |
|  | *Log Odds* | *SE* | *p* | *Log Odds* | *SE* | *p* | *Log Odds* | *SE* | *p* |
| Study 1 (income inequality) | 20.53 | 2.58 | <.001 | -0.79 | 0.35 | .025 | -21.317 | 2.57 | <.001 |
| Study 2 (climate change) | 18.80 | 2.63 | <.001 | -1.42 | 0.31 | <.001 | -20.218 | 2.62 | <.001 |
| Study 3 (climate change) | 19.88 | 2.74 | <.001 | 1.13 | 0.55 | .040 | -18.752 | 2.69 | <.001 |

*Log odds, Standard Errors, and P-Values for Study as a Predictor of Profile Membership
Note*. Study 4 (gun control/rights) was used as the reference category. Analyses were conducted using the full sample (*N* = 509)
